# Cholinergic nucleus degeneration and its association with gait impairment in Parkinson’s disease

**DOI:** 10.1186/s12984-024-01417-7

**Published:** 2024-07-18

**Authors:** Xiaodan Zhang, Mateng Wang, Shi Yeow Lee, Yumei Yue, Zhaoying Chen, Yilin Zhang, Lulu Wang, Qiongfeng Guan, Weinv Fan, Ting Shen

**Affiliations:** 1 Department of Neurology, Ningbo NO.2 Hospital, NO.6 Building, 41 Xibei Street, Haishu District, Ningbo, Zhejiang Province China; 2 Department of Emergency Medicine, The University of Hong Kong, Hong Kong SAR, China; 3 Department of General Surgery, Yinzhou NO.2 Hospital, Ningbo, Zhejiang Province China; 4 Department of Neurology of Sir Run Run Shaw Hospital, Zhejiang University School of Medicine, Zhejiang University, Hangzhou, Zhejiang Province China; 5 Department of Neurology, Perelman School of Medicine, University of Pennsylvania, Philadelphia, PA USA

**Keywords:** Gait, Cholinergic nucleus, Magnetic resonance imaging, Parkinson’s disease, Voxel-based morphometry analysis, Gray matter density, Gray matter volume, Degeneration

## Abstract

**Background:**

The contribution of cholinergic degeneration to gait disturbance in Parkinson’s disease (PD) is increasingly recognized, yet its relationship with dopaminergic-resistant gait parameters has been poorly investigated. We investigated the association between comprehensive gait parameters and cholinergic nucleus degeneration in PD.

**Methods:**

This cross-sectional study enrolled 84 PD patients and 69 controls. All subjects underwent brain structural magnetic resonance imaging to assess the gray matter density (GMD) and volume (GMV) of the cholinergic nuclei (Ch123/Ch4). Gait parameters under single-task (ST) and dual-task (DT) walking tests were acquired using sensor wearables in PD group. We compared cholinergic nucleus morphology and gait performance between groups and examined their association.

**Results:**

PD patients exhibited significantly decreased GMD and GMV of the left Ch4 compared to controls after reaching HY stage > 2. Significant correlations were observed between multiple gait parameters and bilateral Ch123/Ch4. After multiple testing correction, the Ch123/Ch4 degeneration was significantly associated with shorter stride length, lower gait velocity, longer stance phase, smaller ankle toe-off and heel-strike angles under both ST and DT condition. For PD patients with HY stage 1–2, there were no significant degeneration of Ch123/4, and only right side Ch123/Ch4 were corrected with the gait parameters. However, as the disease progressed to HY stage > 2, bilateral Ch123/Ch4 nuclei showed correlations with gait performance, with more extensive significant correlations were observed in the right side.

**Conclusions:**

Our study demonstrated the progressive association between cholinergic nuclei degeneration and gait impairment across different stages of PD, and highlighting the potential lateralization of the cholinergic nuclei’s impact on gait impairment. These findings offer insights for the design and implementation of future clinical trials investigating cholinergic treatments as a promising approach to address gait impairments in PD.

**Supplementary Information:**

The online version contains supplementary material available at 10.1186/s12984-024-01417-7.

## Introduction

Gait disturbances are prevalent and disabling symptoms of Parkinson’s disease (PD), with no medication available to halt their progression [1, 2]. Previous studies have indicated that gait disturbances in PD are primarily caused by dopaminergic dysfunction [1]. Dopaminergic therapy has shown effectiveness in improving gait impairment, particularly in pace-related measures. However, as the disease progresses, it is noted that some measures of gait impairment become unresponsive to dopaminergic treatment [1, 3]. Moreover, certain aspects of gait performance, such as variability of stance time, exhibit resistance to dopamine-based interventions, leading to mobility disturbances that are associated with falls and functional disability [1, 3–5].

Accumulating evidence supports the involvement of cholinergic dysfunction as a non-dopaminergic contributor to gait dysfunction in PD [6, 7]. Consequently, investigating the cholinergic pathways implicated in gait dysfunction holds promise for the development of future therapeutic strategies targeting PD-related gait impairment. Cholinergic degeneration is a common early manifestation of PD, that worsens as the disease progresses [8]. The brain encompasses two major cholinergic projection systems: the cholinergic basal forebrain (cBF) and mesopontine tegmental [8]. The cBF comprises cholinergic nuclei 123 (Ch123) that project to the hippocampus and olfactory system, and Ch4 that projects to the cortex [8]. Extensive research has extensively examined the association between cholinergic mesopontine tegmental dysfunction and gait degeneration in PD [9]. Accordingly, pedunculopontine nucleus (PPN) region deep brain stimulation (DBS) has emerged as a promising therapy for PD. However, a comprehensive literature review suggests that while improvements in gait freezing and reductions in falls have been observed with PPN-DBS, its impact on overall gait remains inconclusive [10]. Recent neuroimaging studies have provided compelling evidence supporting the role of cBF in contributing to gait dysfunction in PD. Specifically, a magnetic resonance imaging (MRI) study has identified significant atrophy in the Ch4 region in PD patients with concurrent freezing of gait (FOG), and the severity of Ch4 atrophy has been found to positively correlate with the severity of FOG [11]. Positron emission tomography-computed tomography (PET-CT) study has further demonstrated a connection between the postural control and the right hemisphere cholinergic network [12]. Cortical cholinergic degeneration has been shown to be associated with slower gait velocity(GV) in PD [6, 13]. Furthermore, recent studies have revealed that Ch4 degeneration is associated with GV and the longitudinal progression of gait impairments in PD [4, 14].

However, the contribution of cholinergic degeneration to comprehensive gait parameters, especially dopaminergic resistant gait parameters remain unclear. The comprehensive gait characteristics include spatiotemporal and kinematic parameters, variability, and symmetry of gait in both single-task (ST) and dual-task (DT) walking. Our previous study suggested that PD patients exhibited a slower GV, a shorter stride length (SL), a shorter swing time, a smaller toe-off (TO) and heel-strike (HS) angles compared with controls in both ST and DT walking [15]. Therefore, it is crucial to investigate the association between cholinergic dysfunction and comprehensive gait parameters to identify new therapeutic approaches for gait dysfunction in PD. The development of wearable gait analysis and neuroimaging technologies have facilitated the understanding of gait disturbance in PD. Sensor-based gait analysis can capture the kinematics and dynamics of gait, thus provide comprehensive quantitative gait parameters [15]. Brain structural analysis of magnetic resonance imaging (MRI) technology enables comprehensive assessment of the morphological features of cBF, encompassing the gray matter density (GMD) and volumes (GMV) for bilateral Ch123 and Ch4 [1, 4, 7, 14].

This study aims to use sensor-based gait analysis and structural MRI to investigate the association between degeneration of bilateral Ch123/Ch4 and comprehensive quantitative gait parameters in PD patients. Our findings will enhance our understanding of the cholinergic contribution to gait dysfunction, identify gait markers for the detection of cholinergic degeneration and provide valuable insights for upcoming clinical trials focusing on cholinergic treatments for gait impairment in PD.

## Methods

### Participants

This cross-sectional study enrolled 84 patients with PD from Ningbo No.2 hospital between March 2020 and April 2022. The inclusion criteria were: (1) diagnosed with PD according to Movement Disorder Society (MDS) criteria [16], (2) patients at Hoehn and Yahr (HY) stage 1–3, (3) able to walk independently. We also included 69 age- and sex-matched healthy controls (HC). The exclusion criteria were: (1) contraindications for 3T MRI scan, (2) non-right-handed participants, (3) inability to follow instructions. All subjects underwent a brain 3T MRI scan. A subset of the cohort, including 79 PD patients completed gait assessment. Due to personal preferences, 5 patients declined participation in gait assessments during their outpatient enrollment. All PD patients were evaluated in the OFF state (a 18 h-withdrawal of the antiparkinsonian medication).

This study was performed in line with the principles of the Declaration of Helsinki. Approval was granted by the ethics committee of the Ningbo No.2 hospital (Date 2020-02-25/No: PJ-NBEY-KY-2020-023-01). Informed consent was obtained from all individual participants included in the study.

### Clinical data collection

Demographic information was collected. We applied Hamilton Anxiety Rating Scale-24 (HAMA-24) and the Hamilton Depression Rating Scale-24 (HMAD-24) to assess anxiety and depression. The Mini-Mental State Examination (MMSE) and Montreal Cognitive Assessment (MoCA) were used to evaluate cognitive functions. Executive functions were examined using visuospatial/executive subscale of MoCA. Movement disorder specialists collected: disease duration, levodopa equivalent daily dose (LEDD), Movement Disorder Society Unified Parkinson’s Disease Rating Scale part III (MDS-UPDRS III), HY stage scale, Berg Balance Scale (BBS), Mini-Balance Evaluation Systems Test (Mini-BEST), Freezing of Gait Questionnaire (FOGQ), Non-Motor Symptoms Questionnaire (NMSQ), and Fatigue Severity Scale (FSS). The ratio of the mean MDS-UPDRS tremor score to the mean MDS-UPDRS postural instability/gait difficulty (PIGD) score was used to categorize tremor dominant (TD) (ratio ≥ 1.15), PIGD (≤ 0.90), and indeterminate (0.9–1.15) phenotypes of PD patients [17].

### Gait evaluation

**Fig. 1:**
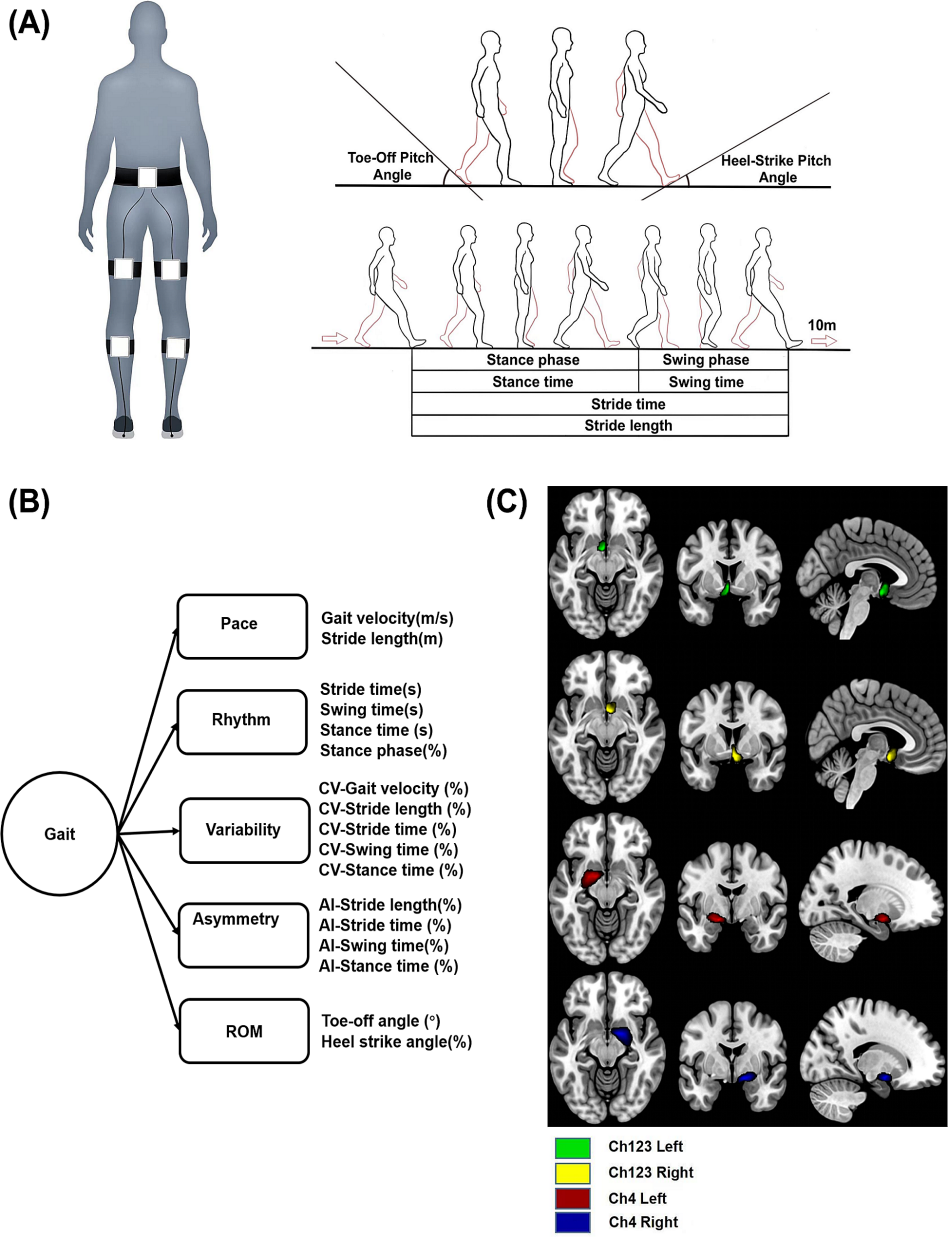
Illustration of Ch123/Ch4 and gait parameters. **(A)** Schematic illustration of gait evaluation. **(B)** Collected gait parameters. **(C)** Visualization of the Ch123 and Ch4 in both hemispheres. Abbreviations: Ch123/Ch4, cholinergic nuclei 123/cholinergic nuclei 4; CV, coefficient of variation; AI, asymmetry index; ROM: range of motion

Gait parameters were collected using the JiBuEn^®^ system, as depicted in Fig. 1a [15, 18]. Participants performed two tests at their self-selected pace:

(1) Single-task (ST) walking test: Participants walked in a straight line on a 10-meter footpath for a distance of 10 m.

(2) DT walking test: Participants simultaneously performed a serial addition task while walking for 10 m. They started with the number 1 and verbally added subsequent numbers (e.g., 2, 3, etc.) to the previous sum, requiring simultaneous engagement in both tasks.

Before the formal walking tests, participants completed one practice trial without data collection with the JiBuEn^®^ system: they were instructed to walk at their preferred speed on a 10-meter trail (ST walking practice), then turn around and walk back along the same 10-meter trail while performing the serial addition task (DT walking practice). After the practice trial, participants performed the formal ST and DT 10-meter walking tests, during which gait parameters were collected using the JiBuEn^®^ system.

Methods to collect gait parameters have been described in our previous study [15]. Gait parameters were obtained from each 10-meter walking test, encompassing both spatiotemporal and kinematic parameters, as shown in Fig. 1b. Spatiotemporal parameters include pace parameters (GV and SL), and rhythm parameters (stride time, swing time, stance time, and stance phase) [19]. Kinematic parameters include the ankle range of motion (ROM) parameters, namely TO and HS [20]. The variability of gait parameters was evaluated using the coefficient of variation (CV), including variability of GV (CV-GV), SL (CV-SL), stride time(CV-Stride time), swing time (CV-Swing time), and stance time (CV-Stance time) [21]. The asymmetry of gait parameters was evaluated using the asymmetry index (AI), including asymmetry of SL (AI-SL), stride time(AI-Stride time), swing time (AI-Swing time), and stance time (AI-Stance time) [22, 23].

### Neuroimaging

All participants underwent a brain MRI scan using a 3 Tesla Siemens Prisma scanner system (Siemens Medical Solutions, Erlangen, Germany) while lying in supine position with head securely fixed using a belt and foam pads to minimize head motion. High-resolution axial images were obtained using 3D T1-weighted magnetization-prepared rapid acquisition gradient echo (MPRAGE) sequence with the following parameters: repetition time/echo time = 1900/2.1 ms, slice thickness/gap = 1.0/0 mm, flip angle = 9°, inversion time = 900 ms, matrix = 256 × 256, field of view = 240 mm × 240 mm, voxel size = 1 × 1 × 1 mm3, and 188 sagittal slices.

Images were preprocessed using FSL (6.0.3) (https://fsl.fmrib.ox.ac.uk/fsl), including motion correction, gradient direction correction, and removal of non-brain tissues. We extracted the GMD and GMV of Ch123/Ch4 according to the previously published methods [4, 14]. The Statistical Parametric Mapping (SPM) 12 software (https://www.fil.ion.ucl.ac.uk/spm/) was imbedded in MATLAB (Mathworks, Natwick, Massachusetts, USA). The Computational Anatomy Toolbox is an extension to SPM12 (CAT12) (http://www.neuro.uni-jena.de/cat/) to perform voxel-based morphometry (VBM) analysis on T1-weighted MPRAGE images. This procedure automatically segmented images into gray matter, white matter, and cerebrospinal fluid. The Diffeomorphic Anatomical Registration Through Exponentiated Lie Algebra (DARTEL) algorithm was applied to register the extracted gray and white matter to the Montreal Neurological Institute (MNI) space in a non-linear manner [24]. The gray matter volumes were modulated using the Jacobian determinant obtained during spatial normalization.

The Ch123 and Ch4 probability maps were used to label the regions of interest (ROI) within the Ch123/Ch4 mask (Fig. 1c), which was resliced to have the same dimensions as the VBM-processed images [25–27]. The Restplus toolbox was used to extract the mean value of density from the ROIs to obtain GMD of Ch123 and Ch4 [28]. The sum of the voxel values within the ROIs was calculated to obtain GMV of Ch123 and Ch4. Total intracranial volume (TIV), which is the sum of gray matter, white matter, and cerebrospinal fluid volumes, was used to normalize the GMV values.

### Statistical methods

R software (R Foundation for Statistical Computing, V4.0.3, Austria) was used to conduct statistical analyses. The comparison of measured data between groups was evaluated by using the independent t-test for normally distributed data, the Mann-Whitney U test for empirical distribution, and the chi-squared test for the categorical data. Multiple linear regression model was employed to investigate the difference of GMD and GMV of Ch123/Ch4 between PD and HCs, as well as correlations between GMD and GMV of Ch123/Ch4, gait parameters, and scale scores in PD group. These statistical analyses were adjusted for age and sex, while the analysis of GMV additionally adjusted for TIV. Shapiro-wilk test was used to check the distribution of the residuals of multiple linear regression models. For those residuals not normally distributed, data log2 or box-cox data transformation were performed. For those gait parameters associated with Ch123/Ch4 degeneration, we proceeded with subgroup analysis and controlling for confounding factors using multiple linear regression models. A significance level of *P* < 0.05 was set for determining statistical significance. Benjamini-Hochberg multiple testing correction with a prespecified false discovery rate (FDR) of 0.05 was applied to correct all *P*-values. For example, for the correlations between gait parameters and cholinergic nucleus regions, we performed 136 multiple linear regressions, and the Benjamini-Hochberg multiple testing correction was used to correct for 136 comparisons. Similarly, for the relationship between scales and cholinergic regions, a total of 152 multiple linear regressions were conducted, and consequently, 152 multiple comparisons were adjusted for using the Benjamini-Hochberg correction.

## Results

### Participants characteristics

A total of 153 individuals were included in this study, comprising 84 PD patients and 69 age- and sex-matched HCs. The characteristics of all participants are presented in Table 1. PD and HC groups did not significantly differ in terms of age, sex, education level, MMSE and MoCA scores. However, PD patients demonstrated significantly higher HAMD score compared to HCs, indicating that PD group may also experience a higher burden of depressive symptoms. PD patients had a poorer performance in pace, rhythm, variability, and asymmetry gait domain under DT condition comparing with ST (Supplement Table 1).

**Table 1:** Demographic and clinical characteristics of PD and control groups

	PD (*n* = 84)	Control (*n* = 69)	*p*
Age (years)	66.2 ± 8.3	64.0 ± 9.8	0.244
Males (%)	44.0 (52.4)	29.0 (42.0)	0.202
Education (years)	6.5 ± 4.6	7.9 ± 5.1	0.176
MMSE	24.5 ± 5.1	26.1 ± 4.0	0.163
HAMD-24	9.7 ± 8.0	5.0 ± 6.7	0.002
HAMA-24	9.3 ± 7.3	-	-
MoCA	20.0 ± 4.9	22.9 ± 6.5	0.155
Visuospatial/Executive	3.2 ± 1.2	3 ± 1.3	0.691
Naming	2.9 ± 0.4	2.4 ± 0.9	0.017
Memory	5.2 ± 1.4	5 ± 1.2	0.598
Attention	1.8 ± 1.2	1.8 ± 0.9	0.819
Language	1.4 ± 0.7	1 ± 0.9	0.310
Abstraction	3 ± 2.1	1.4 ± 1.8	0.033
Delayed Recall	5.4 ± 1.4	5.5 ± 1	0.706
Orientation	3.2 ± 1.2	3 ± 1.3	0.691
Phenotype of PD			
TD (%)	21 (25.0%)	-	-
PIGD (%)	45 (53.6%)	-	-
Indeterminate (%)	18 (21.4%)	-	-
PD patients with FOG(%)	13 (15.5%)		
PD patients without FOG(%)	71 (84.5%)		
Disease duration (months)	54.5 ± 43.9	-	-
LEDD (mg)	350.7 ± 309.3	-	-
MDS-UPDRS III	36.0 ± 16.0	-	-
HY stage	2.1 ± 0.8	-	-
HY 1–2	49 (58.3%)	-	-
HY > 2	35 (41.7%)	-	-
BBS	51.5 ± 7.5	-	-
Mini-BEST	23.47 ± 4.3	-	-
APA	4.0 ± 1.5	-	-
Reactive balance	5.3 ± 1.2	-	-
Sensory	5.6 ± 1.1	-	-
DyG	8.8 ± 1.6	-	-
FOGQ	6.0 ± 8.4	-	-
NMSQ	7.9 ± 4.4	-	-
FSS	2.6 ± 2.3	-	-

Values are expressed as means ± standard deviation for the continuous variables, as frequencies for the categorical variables

PD: Parkinson’s disease; MMSE: Mini-Mental State Examination; HAMD-24: Hamilton Rating Scale for Depression-24; HAMA-24: Hamilton Anxiety Rating Scale-24; MoCA: Montreal Cognitive Assessment; FOG: Freezing of Gait; LEDD: Levodopa equivalent daily dose; MDS-UPDRS III: Movement Disorder Society Unified Parkinson’s Disease Rating Scale part III; HY stage: Hoehn and Yahr stage; BBS: Berg Balance Scale; Mini-BEST: Mini Balance Evaluation Systems Test; APA: Anticipatory postural adjustments; Sensory: Sensorimotor integration; DyG: dynamic gait; FOGQ: Freezing of Gait Questionnaire; NMSQ: Non-Motor Symptoms Questionnaire; FSS: Fatigue Severity Scale

### Comparison of structural signatures of Ch123/Ch4

**Fig. 2:**
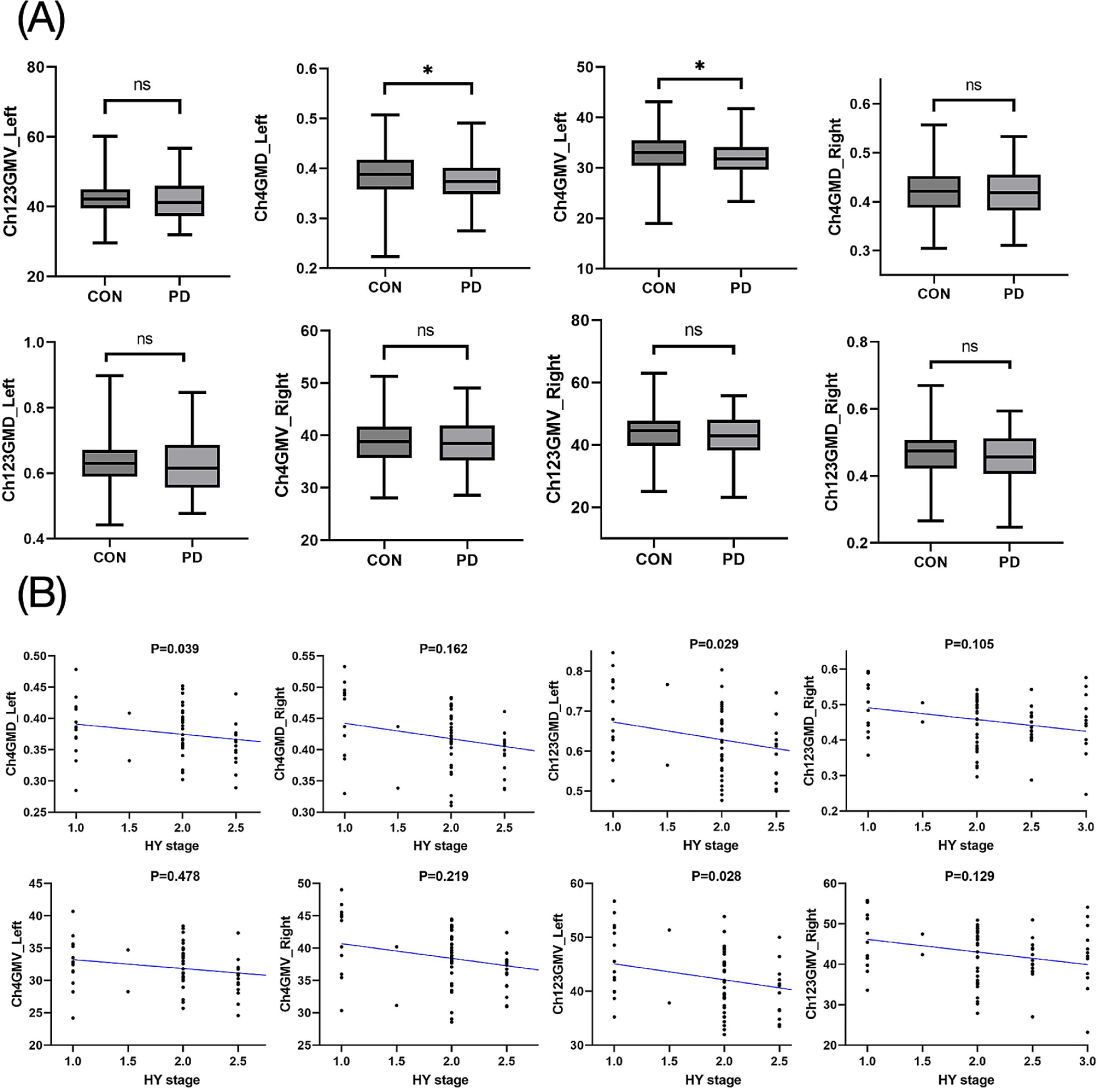
Comparison of structural signatures of Ch123/Ch4 between groups. **(A)** Comparison of GMD and GMV in the Ch123/Ch4 between PD and controls. **(B)** Distribution of GMD and GMV in the Ch123/Ch4 in PD patients across different HY stages. Abbreviations: Ch123/Ch4, cholinergic nuclei 123/cholinergic nuclei 4; GMD, gray matter density; GMV, gray matter volume; PD, Parkinson’s disease; CON, controls; HY stage, Hoehn and Yahr stage

We conducted a thorough analysis of brain structure alterations in bilateral Ch4 and Ch123 in PD. Our results revealed significant differences between PD patients and HCs after adjusting for age and sex in the analysis of GMD, and additionally adjusting for TIV in the analysis of GMV. PD patients showed significant decrease in both GMD and GMV of the left Ch4 compared to HCs (Fig. 2a, Supplemental Table 2). Additionally, although not reaching statistical significance, PD patients showed a tendency towards decreased GMD and GMV in the right Ch4 and bilateral Ch123 compared to HCs.

To gain deeper insights into the progression of brain atrophy, we conducted subgroup analyses within the PD group. No significant differences were observed in the GMD and GMV of the Ch123/Ch4 between patients with PD in HY stage 1–2 and HCs. However, as the disease progressed, there was a notable atrophy in the left Ch4 region among individuals with PD in HY stage > 2 when compared to the HC group, as shown in Supplemental Table 3. A gradual pattern of atrophy was observed in both GMD and GMV of the Ch123/Ch4 as HY stages increased. Significant associations were observed between the GMD and GMV of left Ch123 and HY stages (Fig. 2b, Supplemental Table 4). Moreover, to explore potential laterality in the atrophy of cholinergic nuclei, we compared the left and right sides. Paired T-tests revealed significant differences in the GMD and GMV of the left and right Ch123/Ch4 in PD patients, indicating asymmetry in these nuclei (Supplemental Table 5). Specifically, the left Ch4 showed lower GMV and GMD, and left Ch123 had lower GMV, compared to their counterparts on the right side. Conversely, the right Ch123 exhibited lower GMD compared to the left side. Furthermore, we found no relationship between levodopa equivalent daily dose (LEDD) and the degeneration of these nuclei (Supplemental Table 6).

### Association between structural signatures of Ch123/Ch4 and clinical scales

**Fig. 3:**
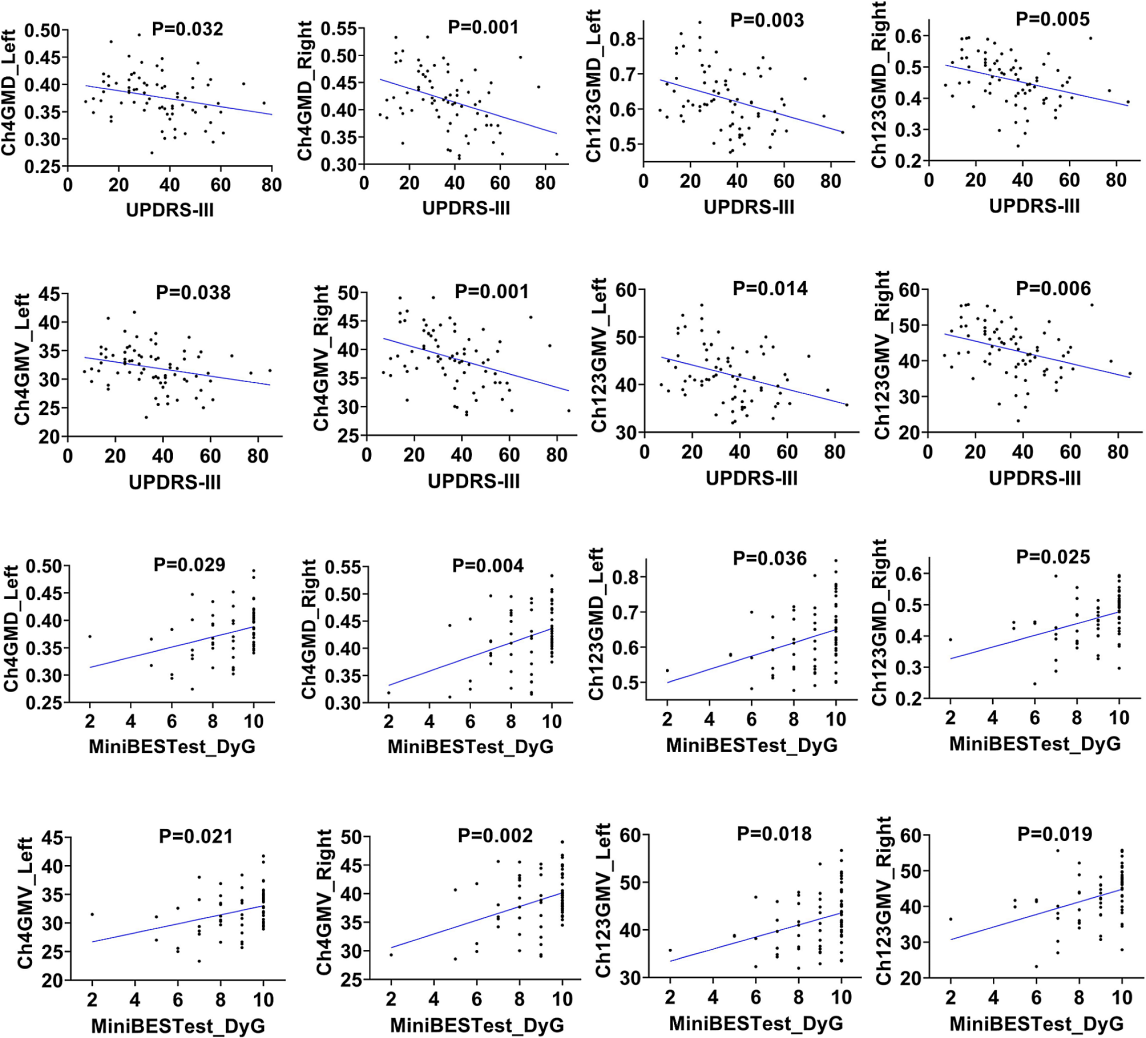
Associations between GMD and GMV of Ch4 and MDS-UPDRS-III, and Mini-BEST_DyG in PD patients. Abbreviations: Ch4, cholinergic nuclei 4; GMD, gray matter density; GMV, gray matter volume; PD, Parkinson’s disease; UDPRS III, Unified Parkinson’s Disease Rating Scale Part III; Mini-BEST_DyG, dynamic gait subscale of Mini Balance Evaluation Systems Test

In our analysis, we explored the association between clinical scales and the GMD and GMV of Ch123/Ch4, while adjusting for age and sex in the analysis of GMD and further adjusting for TIV in the analysis of GMV (Fig. 3, Supplement Table 7). Both GMV and GMD of the Ch123 and Ch4 were correlated with MDS-UPRDRS-III, and dynamic gait (DyG) subscale of Mini-BEST. Additionally, FOGQ score exhibited correlations with GMV of Ch123, as well as both GMV and GMD of Ch4. However, there were no significant correlations found between Ch123/Ch4 degeneration and HAMA, HAMD, or education level adjusted MMSE scores and MoCA scores.

### Association between gait parameters and structural signatures of Ch123/Ch4

We further evaluated the associations between gait parameters and the GMD and GMV of Ch123/Ch4 in PD group, while adjusting for age and sex in the analysis of GMD and additionally adjusting for TIV in the analysis of GMV (Table 2, Supplement Tables 8, 9, 10 and 11). Significant correlations were observed between gait performance and bilateral Ch123/Ch4, while more extensive significant correlations were observed in the right Ch123/Ch4. During the ST walking test, left Ch123/Ch4 was significantly correlated with pace, rhythm, and ankle ROM, and right Ch123/Ch4 associated with all gait domains. In the DT walking test, the left Ch4 was associated with DT-GV and DT-TO, the right Ch4 was associated with SL, GV, TO, while the right Ch123 were significantly correlated with various gait parameters in pace, rhythm, and ankle ROM domains. After multiple testing correction, only the right cBF correlated with SL, GV, stance phase, TO and HS under ST and DT conditions (Table 2).

**Table 2:** Association between gait parameters and structural signatures of Ch123/Ch4 in PD patients

	Gait domain	B	Lower 95%CI	Upper 95%CI	*p*	*p*’
**Right Ch4 GMD**						
ST-GV (m/s)	Pace	1.39	0.45	2.33	0.004	0.039
***Right Ch123 GMD***						
ST-SL (m)*	Pace	0.87	0.29	1.44	0.004	0.039
ST-GV (m/s)	Pace	1.11	0.44	1.78	0.002	0.030
ST-Stance phase	Rhythm	-13.54	-22.73	-4.34	0.004	0.039
ST-TO (°)*	ROM	1382.88	492.19	2273.56	0.003	0.039
DT-SL	Pace	1.11	0.42	1.79	0.002	0.035
DT-GV	Pace	1.31	0.60	2.01	0.001	0.014
DT- Stance phase*	Rhythm	-0.0002	-0.0001	-0.0006	0.003	0.048
DT-TO*	ROM	1691.66	806.23	2566.10	< 0.001	0.014
***Right Ch4 GMV***						
ST-SL (m)*	Pace	0.01	0.01	0.02	0.002	0.030
ST-GV (m/s)	Pace	0.02	0.01	0.03	0.001	0.023
ST-Stance phase	Rhythm	-0.21	-0.35	-0.07	0.004	0.039
ST-TO (°)*	ROM	21.86	8.21	35.51	0.002	0.030
***Right Ch123 GMV***						
ST-SL (m)*	Pace	0.01	0.01	0.02	< 0.001	0.009
ST-GV (m/s)	Pace	0.01	0.01	0.02	< 0.001	0.009
ST-Stance phase	Rhythm	-0.18	-0.28	-0.08	< 0.001	0.016
ST-TO (°)*	ROM	18.59	9.01	28.18	< 0.001	0.009
ST-HS (°)	ROM	0.45	0.21	0.69	< 0.001	0.010
DT-SL	Pace	0.01	0.01	0.02	< 0.001	0.014
DT-GV	Pace	0.01	0.01	0.02	< 0.001	0.014
DT-TO*	ROM	20.72	10.98	30.46	< 0.001	0.002
DT-HS	ROM	0.42	0.17	0.66	0.001	0.005

Multiple linear regression was used to examine the age- and sex-adjusted associations between gait parameters, and the values of GMD, age-, sex- and TIV-adjusted associations between gait parameters and the values of GMV. *, indicated the residuals of multiple linear regression models were not normally distributed, and data transformation was performed. *p’*, *p* values corrected by Benjamini-Hochberg multiple testing correction for 136 comparisons

Ch123/Ch4: cholinergic nuclei 123/ cholinergic nuclei 4; PD: Parkinson’s disease; GMD: gray matter density; ST: single task; SL: stride length; GV: gait velocity; TO: toe-off angle; HS: heel strike angle; ROM: range of motion; DT: dual-task; GMV: gray matter volume; CV: coefficient of variation; AI: asymmetry index; TIV, total intracranial volume

We employed stepwise regression to assess the relative contribution of Ch123/Ch4 to the 10 gait parameters considering the influence of cofounders. Adding UPDRS III, MMSE, HAMD-24, and HAMA-24 scores, or LEDD to the models did not affect the ten gait parameters and Ch123/Ch4 associations, as shown in Supplemental Tables 12–13.

We further conducted a subgroup analysis to examine the relationship between the 10 gait parameters and Ch123/Ch4, as shown in Supplemental Table 14. We observed significant associations between gait parameters and the right Ch123 in non-FOG PD patients. Additionally, in PD patients with left onset, we found that the GMV of the right Ch123 was significantly associated with the ST-stance phase, ST-TO, and DT-TO, after controlling for multiple comparisons.

In PD patients with HY stages 1–2, we revealed that the right Ch123/Ch4 was significantly correlated with SL, GV, TO and HS under ST and DT. Upon multiple testing correction, only DT-GV and DT-TO exhibited significant correlations with the GMV of the right Ch123, as presented in Supplemental Table 15. For PD patients with HY > 2, the GMD of the left Ch4 and Ch123 were only associated with DT-TO, while the right side Ch123/Ch4 correlated with ST-stance phase, SL, GV, TO, and HS under both ST and DT conditions. After multiple testing correction, both ST-SL, ST-GV, ST-stance phase, ST-TO, and ST-HS were found to be significantly associated with the GMD and GMV of the right Ch123, as shown in Supplemental Table 16.

## Discussion

This study is the first to examine the relationship between bilateral Ch123/Ch4 degeneration and comprehensive gait impairment in PD patients. Utilizing MRI morphometric measurement of cholinergic nucleus, we found that PD patients exhibited Ch123/Ch4 degeneration compared to controls. Additionally, using sensor-based gait analysis, we discovered significant associations between Ch123/Ch4 degeneration and impaired pace, rhythm, and ankle ROM of gait. Importantly, these relationships were observed exclusively in the right hemisphere of PD patients after multiple testing correction. In subgroup analysis, for PD with HY 1–2, no atrophy was observed in the Ch123/Ch4 when compared with controls, while right Ch123/Ch4 was significantly correlated with gait performance. With disease progresses, atrophy in the left Ch4 was observed, and significant correlations were found between gait performance and bilateral Ch123/Ch4, with more extensive significant correlations were observed in the right Ch123/Ch4. These results highlighted that right cholinergic nucleus degeneration was closely associated with gait impairment in PD patients.

PD is a multisystem disorder, with growing evidence indicating the involvement of cholinergic system degeneration in its progression [29]. Previous studies have indicated the occurrence of cholinergic degeneration in both cortical and subcortical regions in PD, as evidenced by reduced cholinergic neuron activity [30], impaired microstructural integrity [31], and macrostructural alterations [30, 32, 33]. Moreover, the cholinergic nucleus degeneration might be more pronounced among PD patients in the advanced stages compared to those in the early stages [29, 32]. Consistent with previous studies, we also found significant alterations in morphological measures of Ch123/Ch4 in PD patients, accompanied by a progressive pattern of atrophy in Ch123/Ch4 as the HY stage increased.

Cholinergic nuclei degeneration has been reported to be associated with cognitive impairment in PD, especially in the attention and visuospatial domains [25, 34, 35]. Inconsistent with previous studies, our study observed no significant correlations between the cholinergic nuclei degeneration and various cognitive assessment scales. Only the lower scores in the attention and orientation domain of the MoCA scale showed a tendency to be associated with decreased GMD and GMV in the right Ch4. One possible explanation for this discrepancy is that our study included elderly Chinese individuals, the majority of whom had received only primary education, which could have contributed to difficulties in comprehending the MoCA scale and resulted in lower accuracy in assessing cognitive impairment [36, 37]. In contrast, previous studies were conducted in socioeconomically developed countries, where participants were well-educated with an average education of around 16 years [25, 34, 35]. To further support our hypothesis, both the PD and control groups in our study had mean MoCA scores ranging from 20 to 22, which were lower than those reported in studies conducted in developed countries (typically ranging from 27 to 28) [25, 34, 35]. Therefore, it is crucial to interpret our negative results with caution.

The Ch123/Ch4 nuclei, which have extensive cortical projections, are closely associated with attention, memory, and executive functions, all of which are crucial for gait [38]. Consequently, the degeneration of the cholinergic basal forebrain (cBF) system in Parkinson’s disease (PD) patients can result in gait impairments [38]. Previous animal and in vivo studies have confirmed the significant role of cBF in attention and gait impairments in PD patients [38]. Neuroimaging studies have also demonstrated a notable correlation between cBF degeneration in PD patients and gait impairments, with attentional deficits playing a mediating role [4, 11, 14, 39]. While previous research has predominantly focused on the PPN regarding cholinergic modulation of gait, clinical studies have shown that DBS targeting the PPN for dopaminergic-resistant gait and balance disturbances did not yield effective symptom improvement over a 2-month period [40]. Consequently, cBF may represent a promising novel therapeutic target for developing more effective treatment approaches to address gait impairments in PD patients.

Our study also observed noteworthy correlations between the cholinergic nuclei degeneration and various motor symptom assessment scales. These results are in line with previous studies, and implied that as the atrophy of the nuclei advanced, motor symptoms, particularly gait impairments, progressively worsen [7, 41, 42]. This escalating degeneration along the disease course could potentially serve as a predictive biomarker for disease progression, and potentially pave the way for promising therapeutic strategies that target the cholinergic system [43].

To delve deeper into the relationship between the cholinergic system and gait performance in PD, we employed sensor-based gait analysis, which offers more objective and quantitative gait parameters for capturing subtle gait changes and enhancing sensitivity for early symptom detection and tracking [15, 18]. Our findings contribute additional evidence to emphasize the intricate associations between Ch123/Ch4 degeneration and the pace, rhythm, variability, and ROM domain of gait in PD patients. For instance, the relationship between cholinergic dysfunction and the pace domain in PD has been firmly established in previous studies [4, 6, 13, 14, 44]. In addition to the degeneration of cholinergic neurons in the basal forebrain, the deterioration of the PPN, which houses crucial brainstem cholinergic neurons, is also implicated in gait impairment in PD patients [45]. Recent research has demonstrated that deficits in PPN network connectivity are linked to worsened stride time and stance time, supporting our findings [45]. Furthermore, impaired gait variability and asymmetry have been observed in PD, and these two domains are associated with heightened conscious control and reduced automaticity [46, 47]. Consistent with our finding, a previous study demonstrated the correlation between gait variability and reduced GMV in Ch4 [44]. Notedly, our study is the first to examine the associations between Ch123/Ch4 degeneration and reductions in TO and HS, which are indicators of ankle mobility during walking. In PD patients, early decline in TO and HS increases the risk of tripping over obstacles and ultimately leading to disabilities [15]. Identifying the specific impairments across different gait domains associated with cholinergic degeneration and tailoring targeted rehabilitation interventions could be a potential therapeutic approach.

The degeneration of the dopamine system in PD leads to a disruption in the automaticity of gait [48]. During DT walking, the simultaneous performance of cognitive and walking tasks leads to competition for limited information processing capacity, requiring increased compensatory cognitive control to maintain gait performance [48, 49]. Our study added new evidence that DT gait performance was associated with the degeneration of Ch123 and Ch4. Ch4 projects to the cortex, and numerous studies have demonstrated that cortex degeneration is linked to poorer DT performance [50, 51]. This provides an explanation for the observed connection between Ch4 and DT-GV and DT-TO in our study. On the other hand, Ch123 projects to the hippocampus, primarily contributing to attention and cognitive processes [8]. Interestingly, our study revealed various DT gait parameters associated with Ch123. Notably, a study conducted by Longhurst et al. demonstrated a strong association between cognitive dual-task effects, reduced hippocampal volume, and gait performance [52]. This supports our findings and underscores the significance of cognitive processes in dual-task scenarios. The well-established relationship between gait and cognition implies shared brain circuitry for mobility and cognitive functions [2]. It is conceivable that targeted gait rehabilitation could concurrently address cognitive decline in PD. Previous studies have highlighted the potential of DT training to enhance cognitive function in the elderly [53, 54]. Notably, in PD patients, the DT training not only significantly improves gait performance but also tends to enhance executive function [55, 56]. These findings collectively underscore the role of the cholinergic system in PD, offering therapeutic alternatives beyond pharmacological treatments for addressing PD-related gait impairment and cognitive decline.

Considering that PD exhibits lateralized manifestations, investigating Ch123 and Ch4 in different hemispheres can deepen our understanding of the disease. Our study is the first to examine the associations between comprehensive gait parameters and morphometric measurements of Ch123/Ch4 in bilateral hemispheres. Interestingly, despite reduced GMD and GMV of the left Ch4 compared to the control group, a more pronounced correlation between gait performance and Ch123/Ch4 GMD and GMV was revealed in the right hemisphere, surpassing the significance observed in left hemisphere. Our results are consistent with a PET-CT study that suggests the relatively preserved integrity of the right hemispheric cholinergic system in both PD patients with FOG and non-FOG, while emphasizing the role of the right cortical hemisphere cholinergic system in postural control under challenging conditions [12]. With disease progresses, PD patients with recurrent falls exhibited a loss of integrity in the right brain cholinergic system [57]. These findings together suggested that the perspective right cholinergic system contributes to the gait performance in PD, and loss of integrity of it as the disease progresses contributes to poorer gait impairment. Further supporting our findings, patients with left-sided onset symptoms are more susceptible to experiencing gait abnormalities [58]. Previous MRI studies have also observed structural and functional abnormalities in the right-sided motor circuits in PD patients with freezing of gait [59, 60]. Furthermore, disruptions in the functional connectivity of the right parietal lobe network have been associated with gait abnormalities in PD patients [61]. It also has been observed that the severity of falls was associated with the degeneration of the visual thalamic complex in the right hemisphere [57]. Moreover, when comparing bilateral subthalamic nucleus DBS, it was indicated that right-sided stimulation could result in more significant gait improvements in PD patients [62]. Building upon these studies, our findings further reinforce the lateralization of the right Ch123/4 and its association with gait impairment in PD, thereby lending support to the future design of asymmetric DBS programming.

This study possesses several limitations that warrant acknowledgment. Firstly, the evaluation of gait was conducted in the “off” state, during which both dopaminergic and cholinergic deficiencies are present, potentially introducing complexity to the detection of the relationship between degeneration of Ch123/Ch4 and gait dysfunction. Secondly, our focus on the morphological anomalies of Ch123/Ch4 might not directly reflect cholinergic activity. Thirdly, owing to the limited sample size, this study refrained from performing subgroup analyses across all subgroups within different classifications. We confined to one group within each classification, such as the non-FOG group, and left-onset group. Fourthly, the lack of correlations between gait parameters and neuroimaging findings in healthy subjects limits our ability to establish a direct pathophysiological association solely related to the disease, which should be considered when interpreting the findings. To address these limitations, future studies could benefit from larger PD cohorts and controls, encompassing various HY stages and subgroups. Additionally, turning poses significant challenges for individuals PD and is a common obstacle in their daily walking activities. It is crucial to investigate the underlying mechanisms involved in turning difficulties and identify effective interventions and strategies to alleviate the daily challenges faced by PD patients. Our future study will recruit a larger sample of patients with mid-to-late stage PD and meticulously analyze their gait performance during turning phase to examine the underlying mechanisms contributing to turning difficulties in PD. Besides, employing multimodal neuroimaging assessments would allow for a comprehensive investigation of the independent contributions of dopaminergic and cholinergic deficiencies to gait impairment in PD.

## Conclusion

In conclusion, our study integrated structural neuroimaging and quantitative gait assessment data, to reveal the relationship between cholinergic degeneration and gait impairment in PD. We found that degeneration of Ch123/Ch4 was associated with gait impairments, namely GV, SL, stance phase, TO and HS. Subgroup analysis revealed that atrophy of the left Ch4 was observed in patients with PD after reaching HY stage > 2. Furthermore, in the early stages of PD (HY stage 1–2), there was a significant correlation between gait performance and the Ch123/Ch4 region on the right side. As the disease progressed to HY stage > 2, bilateral Ch123/Ch4 nuclei showed correlations with gait performance, with the correlation being more prominent on the right side. Our findings provide a deeper understanding of the pathogenesis of gait disorders, and offer insights for forthcoming clinical trials exploring cholinergic treatments and personalized gait rehabilitation interventions in addressing gait impairments in PD.

### Electronic supplementary material

Below is the link to the electronic supplementary material.

Supplementary Material 1

## Data Availability

No datasets were generated or analysed during the current study.

## Abbreviations

PD: Parkinson’s disease
GMD: Gray matter density
GMV: Gray matter volume
Ch123/Ch4: Cholinergic nuclei 123/4
ST.: Single task
DT: Dual task
cBF: Cholinergic basal forebrain
GV: Gait velocity
SL: Stride length
TO: Toe-off
HS: Heel-strike
MRI: Magnetic resonance imaging
MDS: Movement Disorder Society
HY: Hoehn and Yahr
HC: Healthy control
HMAD-24: Hamilton Depression Rating Scale-24
HAMA-24: Hamilton Anxiety Rating Scale-24
MMSE: Mini-Mental State Examination
MoCA: Montreal Cognitive Assessment
LEDD: Levodopa equivalent daily dose
MDS-UPDRS: III Movement Disorder Society Unified Parkinson’s Disease Rating Scale part III
BBS: Berg Balance Scale
Mini-BEST: Mini-Balance Evaluation Systems Test
FOGQ: Freezing of Gait Questionnaire
NMSQ: Non-Motor Symptoms Questionnaire
FSS: Fatigue Severity Scale
PIGD: Postural instability/gait difficulty
CV: Coefficient of variation
AI: Asymmetry index
SPM: Statistical Parametric Mapping
VBM: Voxel-based morphometry
DARTEL: Diffeomorphic Anatomical Registration Through Exponentiated Lie Algebra
MNI: Montreal Neurological Institute
ROI: Regions of interest
TIV: Total intracranial volume
FDR: False discovery rate
DyG: Dynamic gait
PPN: Pedunculopontine nucleus
DBS: Deep brain stimulation
