# Supplementary material for: Cholinergic nucleus degeneration and its association with gait impairment in Parkinson’s disease
Source: J Neuroeng Rehabil. 2024 Jul 18;21:120. doi: 10.1186/s12984-024-01417-7 (PMC11256459; doi:10.1186/s12984-024-01417-7)
Supplement: Supplementary file 1 — Supplementary Material 1 [file 12984_2024_1417_MOESM1_ESM.docx]

**Cholinergic nucleus degeneration and its association with gait impairment in Parkinson’s disease**

**Supplementary Table 1.** Single- and dual- task gait parameters in PD group.

|  | Gait domain | ST | DT | *p* | *p’* |
| --- | --- | --- | --- | --- | --- |
| GV (m/s) | Pace | 0.9 ± 0.2 | 0.8 ± 0.2 | 0.008 | **0.017** |
| SL (m) | Pace | 1.0 ± 0.2 | 0.9 ± 0.2 | 0.204 | 0.214 |
| Stride time | Rhythm | 1.1 ± 0.1 | 1.3 ± 0.3 | 0.005 | **0.017** |
| Stance phase | Rhythm | 64.7 ± 2.9 | 66.3 ± 3.4 | 0.006 | **0.017** |
| Stance time | Rhythm | 0.7 ± 0.1 | 0.8 ± 0.2 | 0.125 | 0.146 |
| Swing time | Rhythm | 0.4 ± 0.03 | 0.4 ± 0.1 | 0.083 | 0.116 |
| TO (°) | ROM | 40.5 ± 8.3 | 38.6 ± 8.2 | 0.109 | 0.143 |
| HS (°) | ROM | 26.3 ± 7.6 | 24.4 ± 7.4 | 0.119 | 0.146 |
| CV-GV (%) | Variability | 29.0 ± 8.0 | 31.2 ± 8.4 | 0.277 | 0.277 |
| CV-SL (%) | Variability | 23.2 ± 7.0 | 23.8 ± 5.6 | 0.008 | **0.017** |
| AI-SL (%) | Asymmetry | 24.8 ± 12.9 | 27.4 ± 12.0 | 0.023 | **0.037** |
| CV-stride time (%) | Variability | 24.4 ± 9.3 | 28.2 ± 11.0 | 0.001 | **0.017** |
| AI-stride time (%) | Asymmetry | 24.8 ± 15.7 | 30.6 ± 19.0 | 0.004 | **0.017** |
| CV-swing time (%) | Variability | 22.7 ± 6.9 | 26.1 ± 7.7 | 0.004 | **0.017** |
| AI-swing time (%) | Asymmetry | 25.4 ± 16.6 | 32.8 ± 20.6 | 0.023 | **0.037** |
| CV-stance time (%) | Variability | 16.4 ± 4.3 | 18.2 ± 4.6 | 0.004 | **0.017** |
| AI-stance time (%) | Asymmetry | 13.2 ± 7.7 | 15.8 ± 8.7 | 0.010 | **0.019** |

Note: Values are expressed as means ± standard deviation. *p’, p* values corrected by Benjamini-Hochberg multiple testing correction. Bold values indicate statistical significance, *p* ≤ 0.05.

Abbreviations: PD: Parkinson’s Disease; ST: single-task; DT: dual-task; GV: gait velocity; SL: stride length; TO: toe-off angle; HS: heel strike angle; CV: coefficient of variation; AI: asymmetry index.

**Supplemental Table 2.** Comparison of structural signatures of Ch123/Ch4 between PD and HC groups

|  | B | Lower 95%CI | Upper 95%CI | *p* |
| --- | --- | --- | --- | --- |
| **GMD** |  |  |  |  |
| Left Ch123 GMD | -0.01 | -0.03 | 0.02 | 0.478 |
| Left Ch4 GMD | -0.01 | -0.03 | -0.0004 | **0.043** |
| Right Ch123 GMD | -0.004 | -0.03 | 0.02 | 0.750 |
| Right Ch4 GMD | -0.01 | -0.02 | 0.01 | 0.501 |
| **GMV** |  |  |  |  |
| Left Ch123 GMV | -1.08 | -2.47 | 0.31 | 0.128 |
| Left Ch4 GMV | -1.44 | -2.52 | -0.36 | **0.009** |
| Right Ch123 GMV | -0.75 | -2.73 | 1.23 | 0.457 |
| Right Ch4 GMV | -0.83 | -2.15 | 0.48 | 0.211 |

The comparison of GMD is adjusted for age and gender, comparison of GMV is adjusted for age, gender, and total intracranial volume (TIV). Bold values indicate statistical significance, *p* ≤ 0.05.

Abbreviations: Ch123/Ch4: cholinergic nuclei 123/cholinergic nuclei 4; PD: Parkinson’s Disease; HC: healthy control; GMD: gray matter density; GMV: gray matter volume.

**Supplemental Table 3.** Comparison of structural signatures of Ch123/Ch4 between PD subgroups and HC group

|  | B | Lower 95%CI | Upper 95%CI | *p* |
| --- | --- | --- | --- | --- |
| **PD with HY 1-2 vs. HC** | | | | |
| **GMD** |  |  |  |  |
| Left Ch123 GMD | -0.004 | -0.03 | 0.03 | 0.784 |
| Left Ch4 GMD | -0.01 | -0.03 | 0.007 | 0.253 |
| Right Ch123 GMD | -0.002 | -0.03 | 0.02 | 0.851 |
| Right Ch4 GMD | -0.0004 | -0.02 | 0.02 | 0.959 |
| **GMV** |  |  |  |  |
| Left Ch123 GMV | -0.88 | -2.51 | 0.75 | 0.288 |
| Left Ch4 GMV | -1.08 | -2.33 | 0.17 | 0.090 |
| Right Ch123 GMV | -0.73 | -3.01 | 1.55 | 0.528 |
| Right Ch4 GMV | -0.44 | -1.83 | 0.96 | 0.536 |
| **PD with HY >2 vs. HC** | | | | |
| **GMD** |  |  |  |  |
| Left Ch123 GMD | -0.02 | -0.05 | 0.01 | 0.233 |
| Left Ch4 GMD | -0.02 | -0.04 | -0.003 | **0.022** |
| Right Ch123 GMD | -0.01 | -0.04 | 0.02 | 0.622 |
| Right Ch4 GMD | -0.01 | -0.03 | 0.006 | 0.162 |
| **GMV** |  |  |  |  |
| Left Ch123 GMV | -1.65 | -3.35 | 0.05 | 0.056 |
| Left Ch4 GMV | -2.11 | -3.53 | -0.69 | **0.004** |
| Right Ch123 GMV | -1.04 | -3.62 | 1.54 | 0.424 |
| Right Ch4 GMV | -1.59 | -3.28 | 0.09 | 0.064 |

The comparison of GMD is adjusted for age and gender, comparison of GMV is adjusted for age, gender, and total intracranial volume (TIV). Early stage PD is PD patient at HY stage 1-2. Bold values indicate statistical significance, *p* ≤ 0.05.

Abbreviations: Ch123/Ch4: cholinergic nuclei 123/cholinergic nuclei 4; PD: Parkinson’s Disease; HC: healthy control; GMD: gray matter density; GMV: gray matter volume.

**Supplemental Table 4.** Association between HY stages and structural signatures of Ch123/Ch4 in PD patients

|  | B | Lower 95%CI | Upper 95%CI | *p* |
| --- | --- | --- | --- | --- |
| **GMD** |  |  |  |  |
| Left Ch123 GMD | -2.46 | -4.74 | -0.17 | **0.039** |
| Left Ch4 GMD | -1.80 | -6.18 | 2.59 | 0.425 |
| Right Ch123 GMD | -1.62 | -4.24 | 1.00 | 0.229 |
| Right Ch4 GMD | -2.92 | -6.36 | 0.52 | 0.101 |
| **GMV** |  |  |  |  |
| Left Ch123 GMV | -0.04 | -0.08 | -0.01 | **0.026** |
| Left Ch4 GMV | -0.02 | -0.08 | 0.03 | 0.431 |
| Right Ch123 GMV | -0.02 | -0.05 | 0.01 | 0.224 |
| Right Ch4 GMV | -0.03 | -0.07 | 0.01 | 0.097 |

The comparison of GMD is adjusted for age and gender, comparison of GMV is adjusted for age, gender, and total intracranial volume (TIV). Bold values indicate statistical significance, *p* ≤ 0.05.

Abbreviations: HY stage: Hoehn and Yahr stage; Ch123/Ch4: cholinergic nuclei 123/ cholinergic nuclei 4; PD: Parkinson’s Disease; GMD: gray matter density; GMV: gray matter volume.

**Supplemental Table 5.** Comparison of structural signatures of left and right side Ch123/Ch4 in PD patients.

|  | B | Lower 95%CI | Upper 95%CI | *p* |
| --- | --- | --- | --- | --- |
| Left vs Right Ch123 GMD | 0.17 | 0.16 | 0.18 | **<0.001** |
| Left vs Right Ch4 GMD | -0.04 | -0.05 | -0.04 | **<0.001** |
| Left vs Right Ch123 GMV | -1.05 | -2.09 | -0.01 | **0.024** |
| Left vs Right Ch4 GMV | -6.50 | -7.16 | -5.84 | **<0.001** |

Paired t-test was used to compare GMD and GMV in the left and right side Ch123/Ch4 in PD patients. Bold values indicate statistical significance, *p* ≤ 0.05.

Abbreviations: Ch123/Ch4: cholinergic nuclei 123/ cholinergic nuclei 4; PD: Parkinson’s Disease; GMD: gray matter density; GMV: gray matter volume.

**Supplemental Table 6.** Association between LEDD and structural signatures of Ch123/Ch4 in PD patients

|  | B | Lower 95%CI | Upper 95%CI | *p* |
| --- | --- | --- | --- | --- |
| **GMD** |  |  |  |  |
| Left Ch123 GMD | <-0.01 | -0.004 | 0.0005 | 0.493 |
| Left Ch4 GMD | <-0.01 | -0.00005 | 0.00003 | 0.561 |
| Right Ch123 GMD | <-0.01 | -0.00007 | 0.00005 | 0.818 |
| Right Ch4 GMD | <0.01 | -0.00004 | 0.00005 | 0.912 |
| **GMV** |  |  |  |  |
| Left Ch123 GMV | -0.001 | -0.005 | 0.003 | 0.586 |
| Left Ch4 GMV | -0.001 | -0.004 | 0.002 | 0.642 |
| Right Ch123 GMV | <-0.01 | -0.006 | 0.005 | 0.920 |
| Right Ch4 GMV | <0.01 | -0.004 | 0.004 | 0.828 |

The comparison of GMD is adjusted for age, gender and duration of disease, comparison of GMV is adjusted for age, gender, duration of disease, and total intracranial volume (TIV).

Abbreviations: HY stage: Hoehn and Yahr stage; Ch123/Ch4: cholinergic nuclei 123/ cholinergic nuclei 4; PD: Parkinson’s Disease; GMD: gray matter density; GMV: gray matter volume.

**Supplemental Table 7.** Association between scales and structural signatures of Ch123/Ch4 in PD patients

|  | Ch4 | | | Ch123 | | |
| --- | --- | --- | --- | --- | --- | --- |
|  | B (95%CI) | *p* | *p’* | B (95%CI) | *p* | *p’* |
| **Left GMV** |  |  |  |  |  |  |
| MMSE | 0.18 (-0.17, 0.52) | 0.325 | 0.651 | 0.15 (-0.09, 0.38) | 0.238 | 0.548 |
| MoCA | 0.01 (-0.44, 0.45) | 0.976 | 0.982 | 0.05 (-0.26, 0.35) | 0.777 | 0.918 |
| Visuospatial/Executive | 0.04 (-0.06, 0.16) | 0.401 | 0.726 | 0.01 (-0.06, 0.09) | 0.680 | 0.891 |
| Naming | -0.03 (-0.12, 0.06) | 0.543 | 0.864 | -0.01 (-0.07, 0.05) | 0.708 | 0.908 |
| Attention | 0.06 (-0.05, 0.17) | 0.258 | 0.552 | 0.04 (-0.02, 0.11) | 0.185 | 0.454 |
| Language | -0.09 (-0.179, 0.002) | 0.056 | 0.213 | -0.01 (-0.07, 0.05) | 0.854 | 0.947 |
| Abstraction | -0.03 (-0.11, 0.06) | 0.520 | 0.864 | -0.003 (-0.06, 0.05) | 0.914 | 0.947 |
| Delayed Recall | -0.03 (-0.21, 0.16) | 0.785 | 0.918 | 0.005 (-0.11, 0.12) | 0.927 | 0.947 |
| Orientation | 0.08 (-0.01, 0.18) | 0.087 | 0.276 | 0.01 (-0.05, 0.08) | 0.646 | 0.885 |
| MDS-UPDRS III | -1.27 (-2.47, -0.08) | **0.038** | 0.181 | -0.32 (-0.58, -0.07) | **0.014** | 0.145 |
| FOGQ | -1.08 (-1.96, -0.20) | **0.017** | 0.145 | -0.74 (-1.31, -0.17) | **0.013** | 0.145 |
| BBS | 0.54 (0.003, 1.09) | **0.049** | 0.213 | 0.45 (0.08, 0.83) | **0.019** | 0.145 |
| MiniBEST | 0.27 (-0.05, 0.59) | 0.092 | 0.280 | 0.24 (0.03, 0.46) | **0.029** | 0.176 |
| APA | 0.13 (0.02, 0.25) | 0.020 | 0.145 | 0.10 (0.03, 0.18) | **0.010** | 0.145 |
| Reactive balance | -0.02 (-0.12, 0.07) | 0.656 | 0.888 | 0.003 (-0.07, 0.07) | 0.937 | 0.949 |
| Sensory | 0.02 (-0.06, 0.11) | 0.580 | 0.884 | 0.04 (-0.02, 0.10) | 0.154 | 0.404 |
| DyG | 0.14 (0.02, 0.26) | 0.021 | 0.145 | 0.10 (0.02, 0.19) | **0.018** | 0.145 |
| HAMD-24 | -0.30 (-0.92, 0.31) | 0.330 | 0.651 | -0.27 (-0.69, 0.15) | 0.207 | 0.499 |
| HAMA-24 | 0.03 (-0.52, 0.58) | 0.918 | 0.947 | -0.18 (-0.56, 0.19) | 0.334 | 0.651 |
| **Right GMV** |  |  |  |  |  |  |
| MMSE | 0.19 (-0.06, 0.44) | 0.143 | 0.381 | 0.01 (-0.17, 0.20) | 0.905 | 0.947 |
| MoCA | 0.14 (-0.19, 0.48) | 0.407 | 0.728 | -0.10 (-0.34, 0.13) | 0.384 | 0.712 |
| Visuospatial/Executive | 0.02 (-0.06, 0.11) | 0.557 | 0.864 | 0.01 (-0.05, 0.07) | 0.737 | 0.908 |
| Naming | -0.03 (-0.09, 0.04) | 0.361 | 0.686 | -0.04 (-0.08, 0.01) | 0.107 | 0.296 |
| Attention | 0.07 (-0.01, 0.15) | 0.097 | 0.284 | 0.003 (-0.06, 0.06) | 0.922 | 0.947 |
| Language | 0.01 (-0.06, 0.08) | 0.734 | 0.908 | -0.03 (-0.08, 0.02) | 0.249 | 0.552 |
| Abstraction | 0.02 (-0.05, 0.08) | 0.629 | 0.884 | 0.01 (-0.03, 0.06) | 0.555 | 0.864 |
| Delayed Recall | 0.01 (-0.13, 0.15) | 0.924 | 0.947 | -0.04 (-0.13, 0.06) | 0.464 | 0.811 |
| Orientation | 0.05 (-0.02, 0.12) | 0.183 | 0.454 | -0.01 (-0.06, 0.04) | 0.759 | 0.908 |
| MDS-UPDRS III | -1.40 (-2.22, -0.58) | **0.001** | 0.076 | -0.89 (-1.52, -0.27) | **0.006** | 0.114 |
| FOGQ | -0.86 (-1.46, -0.25) | **0.006** | 0.114 | -0.47 (-0.90, -0.04) | **0.033** | 0.177 |
| BBS | 0.46 (0.08, 0.85) | **0.018** | 0.145 | 0.11 (-0.19, 0.40) | 0.470 | 0.812 |
| MiniBEST | 0.24 (0.02, 0.47) | **0.035** | 0.177 | 0.16 (-0.02, 0.33) | 0.073 | 0.262 |
| APA | 0.08 (0.001, 0.17) | **0.047** | 0.210 | 0.06 (-0.001, 0.12) | 0.053 | 0.213 |
| Reactive balance | 0.02 (-0.05, 0.09) | 0.660 | 0.888 | -0.002 (-0.06, 0.05) | 0.928 | 0.947 |
| Sensory | 0.02 (-0.04, 0.08) | 0.542 | 0.864 | 0.01 (-0.04, 0.05) | 0.755 | 0.908 |
| DyG | 0.14 (0.05, 0.22) | **0.002** | 0.101 | 0.08 (0.01, 0.14) | **0.019** | 0.145 |
| HAMD-24 | -0.11 (-0.56, 0.33) | 0.611 | 0.884 | -0.18 (-0.51, 0.15) | 0.276 | 0.575 |
| HAMA-24 | -0.02 (-0.41, 0.37) | 0.906 | 0.947 | -0.07 (-0.36, 0.22) | 0.627 | 0.884 |
| **Left GMD** |  |  |  |  |  |  |
| MMSE | 18.62 (-10.71, 47.96) | 0.218 | 0.518 | 13.11 (-1.84, 28.05) | 0.091 | 0.280 |
| MoCA | 3.53 (-31.95, 39.00) | 0.847 | 0.947 | 4.77 (-13.08, 22.63) | 0.603 | 0.884 |
| Visuospatial/Executive | 3.95 (-5.48, 13.39) | 0.401 | 0.726 | 0.97 (-3.76, 5.70) | 0.680 | 0.891 |
| Naming | -2.33 (-10.05, 5.39) | 0.543 | 0.864 | -0.71 (-4.57, 3.14) | 0.708 | 0.908 |
| Attention | 5.24 (-4.02, 14.51) | 0.258 | 0.552 | 3.05 (-1.53, 7.63) | 0.185 | 0.454 |
| Language | -7.49 (-15.20, 0.21) | 0.056 | 0.213 | -0.37 (-4.41, 3.67) | 0.854 | 0.947 |
| Abstraction | -2.35 (-9.71, 5.00) | 0.520 | 0.864 | -0.20 (-3.88, 3.48) | 0.914 | 0.947 |
| Delayed Recall | -2.15 (-18.05, 13.75) | 0.785 | 0.918 | 0.36 (-7.56, 8.28) | 0.927 | 0.947 |
| Orientation | 7.15 (-1.10, 15.39) | 0.087 | 0.276 | 0.97 (-3.29, 5.24) | 0.646 | 0.885 |
| MDS-UPDRS III | -105.90 (-202.53, -9.27) | **0.032** | 0.177 | -73.66 (-121.84, -25.49) | **0.003** | 0.114 |
| FOGQ | -90.18 (-166.06, -14.3) | **0.021** | 0.145 | -34.48 (-72.42, 3.45) | 0.074 | 0.262 |
| BBS | 35.45 (-9.06, 79.97) | 0.117 | 0.318 | 18.94 (-4.01, 41.90) | 0.104 | 0.296 |
| MiniBEST | 22.19 (-3.30, 47.68) | 0.087 | 0.276 | 14.00 (0.97, 27.03) | **0.036** | 0.177 |
| APA | 10.66 (1.68, 19.63) | **0.021** | 0.145 | 5.96 (1.25, 10.67) | **0.014** | 0.145 |
| Reactive balance | -1.28 (-8.99, 6.43) | 0.741 | 0.908 | 0.43 (-3.64, 4.50) | 0.835 | 0.947 |
| Sensory | 1.44 (-5.33, 8.20) | 0.673 | 0.891 | 2.03 (-1.50, 5.57) | 0.255 | 0.552 |
| DyG | 10.73 (1.14, 20.33) | **0.029** | 0.176 | 5.46 (0.38, 10.53) | **0.036** | 0.177 |
| HAMD-24 | -25.09 (-75.28, 25.09) | 0.322 | 0.651 | -15.90 (-41.62, 9.82) | 0.222 | 0.519 |
| HAMA-24 | 2.05 (-42.70, 46.79) | 0.927 | 0.947 | -10.27 (-32.90, 12.35) | 0.368 | 0.691 |
| **Right GMD** |  |  |  |  |  |  |
| MMSE | 20.53 (-2.29, 43.36) | 0.083 | 0.276 | 4.60 (-12.61, 21.80) | 0.602 | 0.884 |
| MoCA | 14.45 (-14.15, 43.05) | 0.329 | 0.651 | -7.36 (-28.13, 13.40) | 0.492 | 0.840 |
| Visuospatial/Executive | 2.22 (-5.38, 4.58) | 0.557 | 0.864 | 0.92 (-4.58, 6.42) | 0.737 | 0.908 |
| Naming | -2.81 (-8.95, 3.34) | 0.361 | 0.686 | -3.52 (-7.84, 0.80) | 0.107 | 0.296 |
| Attention | 6.12 (-1.16, 13.39) | 0.097 | 0.284 | 0.26 (-5.19, 5.72) | 0.922 | 0.947 |
| Language | 1.10 (-5.40, 7.60) | 0.734 | 0.908 | -2.66 (-7.27, 1.94) | 0.249 | 0.552 |
| Abstraction | 1.42 (-4.49, 7.33) | 0.629 | 0.884 | 1.25 (-3.00, 5.51) | 0.555 | 0.864 |
| Delayed Recall | 0.61 (-12.15, 13.37) | 0.924 | 0.947 | -3.33 (-12.46, 5.80) | 0.464 | 0.811 |
| Orientation | 4.50 (-2.23, 11.22) | 0.183 | 0.454 | -0.76 (-5.72, 4.21) | 0.759 | 0.908 |
| MDS-UPDRS III | -122.99 (-194.67, -51.30) | **0.001** | 0.076 | -79.76 (-134.74, -24.77) | **0.005** | 0.114 |
| FOGQ | -75.63 (-132.06, -19.20) | **0.010** | 0.145 | -36.28 (-76.98, 4.42) | 0.079 | 0.273 |
| BBS | 33.54 (-0.61, 67.70) | 0.054 | 0.213 | 4.67 (-21.81, 31.16) | 0.726 | 0.908 |
| MiniBEST | 21.15 (1.56, 40.75) | **0.035** | 0.177 | 13.84 (-1.21, 28.90) | 0.071 | 0.262 |
| APA | 7.28 (0.11, 14.46) | **0.047** | 0.210 | 5.52 (-0.04, 11.09) | 0.052 | 0.213 |
| Reactive balance | 1.59 (-4.50, 7.68) | 0.604 | 0.884 | 0.01 (-4.72, 4.73) | 0.998 | 0.998 |
| Sensory | 1.28 (-4.06, 6.62) | 0.634 | 0.884 | 0.42 (-3.73, 4.56) | 0.841 | 0.947 |
| DyG | 11.15 (3.80, 18.51) | **0.004** | 0.114 | 6.73 (0.86, 12.59) | **0.025** | 0.165 |
| HAMD-24 | -10.57 (-49.80, 28.66) | 0.592 | 0.884 | -16.16 (-45.35, 13.02) | 0.273 | 0.575 |
| HAMA-24 | -2.16 (-36.68, 32.37) | 0.901 | 0.947 | -6.22 (-31.94, 19.51) | 0.631 | 0.884 |

Multiple linear regression was used to examine the age- and sex-adjusted associations between scales and the values of GMD, age-, sex- and total intracranial volume (TIV)-adjusted associations between scales and the values of GMV. The MESS and MoCA scores were additionally adjusted with educational level. *p*’*,* *p* values corrected by Benjamini-Hochberg multiple testing correction for 136 comparisons. Bold values indicate statistical significance: *p, p’* ≤ 0.05.

Abbreviations: Ch123/Ch4: cholinergic nuclei 123/ cholinergic nuclei 4; PD: Parkinson’s disease; GMV: gray matter volume; GMV: gray matter volume; MMSE: Mini-Mental State Examination; MoCA: Montreal Cognitive Assessment; MDS-UPDRS III: Movement Disorder Society Unified Parkinson’s Disease Rating Scale part III; FOGQ: Freezing of Gait Questionnaire; BBS: Berg Balance Scale; MiniBEST: Mini Balance Evaluation Systems Test; APA: Anticipatory postural adjustments; Sensory: Sensorimotor integration; DyG: dynamic gait; HAMD-24: Hamilton Rating Scale for Depression-24; HAMA-24: Hamilton Anxiety Rating Scale-24.

**Supplemental Table 8.** Association between gait parameters and GMD of Ch4 in PD patients

|  | ST | | | DT | | |
| --- | --- | --- | --- | --- | --- | --- |
|  | B (95%CI) | *p* | *p*’ | B (95%CI) | *p* | *p*’ |
| **Left Ch4 GMD** |  |  |  |  |  |  |
| SL (m) | 0.89 (-0.16, 1.93) * | 0.095 | 0.250 | 0.99 (-0.24, 2.22) | 0.112 | 0.401 |
| GV (m/s) | 1.35 (0.13, 2.57) | **0.031** | 0.150 | 1.46 (0.18, 2.74) | **0.026** | 0.248 |
| Stance phase | -13.87 (-30.53, 2.80) | 0.102 | 0.250 | -0.0001(-0.0001, 0.0001) * | 0.053 | 0.267 |
| Stride time * | -0.51 (-1.08, 0.05) | 0.075 | 0.217 | -0.66 (-1.47, -0.15) | 0.110 | 0.401 |
| Stance time * | -0.97 (-2.02, 0.09) | 0.071 | 0.210 | -1.34 (-2.91, -0.23) | 0.093 | 0.361 |
| Swing time | -0.07 (-0.25, 0.10) | 0.409 | 0.614 | -0.60 (-1.94, 0.73) | 0.371 | 0.766 |
| TO (°) * | 1373.19 (-249.80, 2996.19) | 0.096 | 0.250 | 1574.98 (-46.79, 3196.74) | 0.057 | 0.277 |
| HS (°) | 13.54 (-27.94, 55.02) | 0.518 | 0.712 | 8.47 (-32.48, 49.42) | 0.681 | 0.862 |
| CV-GV (%) * | 0.01 (-0.17, 0.20) | 0.888 | 0.929 | -0.001 (-0.05, 0.05) | 0.960 | 0.974 |
| CV-SL (%) * | -0.02 (-0.10, 0.06) | 0.558 | 0.751 | -0.01 (-0.11, 0.09) | 0.888 | 0.930 |
| AI-SL (%) * | -0.61 (-3.31, 2.10) | 0.655 | 0.817 | -0.24 (-1.12, 0.64) | 0.589 | 0.831 |
| CV-stride time (%) * | -0.002 (-0.021, 0.018) | 0.865 | 0.919 | -0.03 (-0.18, 0.12) | 0.667 | 0.862 |
| AI-stride time (%) * | -0.003 (-0.317, 0.312) | 0.987 | 0.987 | -1.30 (-4.88, 2.27) | 0.470 | 0.806 |
| CV-swing time (%) * | 0.003 (-0.013, 0.020) | 0.678 | 0.831 | -0.001 (-0.01, 0.01) | 0.742 | 0.877 |
| AI-swing time (%) * | 0.38 (-0.69, 1.45) | 0.479 | 0.693 | -0.06 (-0.21, 0.01) | 0.886 | 0.930 |
| CV-stance time (%) * | 0.006(-0.012, 0.024) | 0.487 | 0.697 | 0.0001 (-0.005, 0.01) | 0.886 | 0.930 |
| AI-stance time (%) * | 0.649 (-0.56, 1.86) | 0.290 | 0.518 | -0.08 (-0.32, 0.16) | 0.514 | 0.826 |
| **Right Ch4 GMD** |  |  |  |  |  |  |
| SL (m) | 1.07 (0.27, 1.88) * | **0.010** | 0.076 | 1.01 (0.03, 1.99) | **0.044** | 0.267 |
| GV (m/s) | 1.39 (0.45, 2.33) | **0.004** | **0.039** | 1.15 (0.12, 2.19) | **0.030** | 0.248 |
| Stance phase | -16.33 (-29.22, -3.45) | **0.014** | 0.095 | -0.0001 (-0.0001, 0.0001) * | 0.067 | 0.314 |
| Stride time * | -0.46 (-0.90, -0.01) | **0.045** | 0.164 | -0.42 (1.08, 0.24) | 0.206 | 0.571 |
| Stance time * | -0.91(-1.73, -0.08) | **0.032** | 0.150 | -0.89 (-2.17, 0.39) | 0.171 | 0.506 |
| Swing time | -0.01 (-0.15, 0.14) | 0.939 | 0.960 | -0.18 (-1.27, 0.90) | 0.737 | 0.877 |
| TO (°) * | 1677.36(427.83, 2926.89) | **0.009** | 0.076 | 1435.34 (133.64, 2737.05) | **0.031** | 0.248 |
| HS (°) | 31.34 (-0.77, 63.44) | 0.056 | 0.186 | 17.03 (-15.87, 49.93) | 0.306 | 0.694 |
| CV-GV (%) * | -0.10 (-0.24, 0.05) | 0.173 | 0.368 | -0.01 (-0.05, 0.03) | 0.574 | 0.831 |
| CV-SL (%) * | -0.05 (-0.12, -0.01) | 0.099 | 0.250 | -0.05 (-0.13, 0.03) | 0.235 | 0.603 |
| AI-SL (%) * | -1.74 (-3.84, 0.36) | 0.103 | 0.250 | -0.54 (-1.24, 0.16) | 0.129 | 0.418 |
| CV-stride time (%) * | -0.01 (-0.02, 0.01) | 0.335 | 0.570 | -0.04 (-0.17, 0.08) | 0.469 | 0.806 |
| AI-stride time (%) * | -0.07 (-0.32, 0.176) | 0.194 | 0.382 | -1.24 (-4.12, 1.65) | 0.397 | 0.766 |
| CV-swing time (%) * | -0.01 (-0.02, 0.01) | 0.224 | 0.429 | -0.002 (-0.01, 0.003) | 0.387 | 0.766 |
| AI-swing time (%) * | -0.22 (-1.07, 0.63) | 0.609 | 0.778 | -0.07 (-0.19, 0.04) | 0.210 | 0.571 |
| CV-stance time (%) * | -0.01 (-0.02, 0.01) | 0.398 | 0.614 | -0.001 (-0.01, 0.003) | 0.715 | 0.876 |
| AI-stance time (%) * | -0.10 (-1.06, 0.87) | 0.845 | 0.909 | -0.10 (-0.29, 0.10) | 0.317 | 0.707 |

Multiple linear regression was used to examine the age- and sex-adjusted associations between gait parameters: and the values of GMD. *, indicated the residuals of multiple linear regression models were not normally distributed, and data transformation was performed. *p*’*,* *p* values corrected by Benjamini-Hochberg multiple testing correction for 136 comparisons. Bold values indicate statistical significance: *p, p’* ≤ 0.05.

Abbreviations: Ch4: cholinergic nuclei 4; PD: Parkinson’s disease; GMD: gray matter density; ST: single task; DT: dual-task; SL: stride length; GV: gait velocity; TO: toe-off angle; HS: heel strike angle; CV: coefficient of variation; AI: asymmetry index.

**Supplemental Table 9.** Association between gait parameters and GMD of Ch123 in PD patients

|  | ST | | | DT | | |
| --- | --- | --- | --- | --- | --- | --- |
|  | B (95%CI) | *p* | *p*’ | B (95%CI) | *p* | *p*’ |
| **Left Ch123 GMD** |  |  |  |  |  |  |
| SL (m) | 0.10 (-0.45, 0.66) * | 0.709 | 0.847 | -0.03 (-0.68, 0.63) | 0.936 | 0.957 |
| GV (m/s) | 0.44 (-0.21, 1.08) | 0.184 | 0.373 | 0.23 (-0.46, 0.92) | 0.513 | 0.826 |
| Stance phase | -5.66 (-14.39, 3.08) | 0.201 | 0.391 | -0.0001 (-0.0001, 0.0002) * | 0.847 | 0.930 |
| Stride time * | -0.32 (-0.61, -0.03) | **0.034** | 0.154 | -0.23 (-0.66, 0.20) | 0.297 | 0.685 |
| Stance time * | -0.55 (-1.10, -0.01) | **0.047** | 0.164 | -0.37 (-1.21, 0.47) | 0.383 | 0.766 |
| Swing time | -0.08 (-0.18, 0.01) | 0.070 | 0.210 | -0.46 (-1.16, 0.24) | 0.192 | 0.544 |
| TO (°) * | 347.38 (-509.64, -1204.40) | 0.422 | 0.624 | 227.02 (-646.18, 1100.23) | 0.606 | 0.832 |
| HS (°) | 4.18 (-17.46, 25.81) | 0.702 | 0.847 | -4.09 (-25.63, 17.45) | 0.706 | 0.873 |
| CV-GV (%) * | -0.02 (-0.113, 0.079) | 0.728 | 0.847 | 0.01 (-0.02, 0.04) | 0.420 | 0.772 |
| CV-SL (%) * | -0.002 (-0.04, 0.04) | 0.916 | 0.944 | 0.01 (-0.04, 0.07) | 0.599 | 0.831 |
| AI-SL (%) * | 0.04 (-1.37, 1.45) | 0.954 | 0.968 | 0.13 (-0.33, 0.59) | 0.572 | 0.831 |
| CV-stride time (%) * | 0.001 (-0.01, 0.01) | 0.786 | 0.883 | 0.04 (-0.04, 0.12) | 0.282 | 0.661 |
| AI-stride time (%) * | 0.08 (-0.08, 0.24) | 0.333 | 0.570 | 0.70 (-1.18, 2.58) | 0.459 | 0.806 |
| CV-swing time (%) * | -0.002 (-0.01, 0.01) | 0.670 | 0.828 | 0.001 (-0.002, 0.004) | 0.467 | 0.806 |
| AI-swing time (%) * | 0.38 (-0.17, 0.93) | 0.171 | 0.368 | 0.03 (-0.05, 0.10) | 0.481 | 0.808 |
| CV-stance time (%) * | 0.003 (-0.01, 0.01) | 0.563 | 0.751 | 0.001 (-0.001, 0.004) | 0.356 | 0.766 |
| AI-stance time (%) * | 0.50 (-0.12, 1.13) | 0.128 | 0.290 | 0.04 (-0.09, 0.17) | 0.560 | 0.831 |
| **Right Ch123 GMD** |  |  |  |  |  |  |
| SL (m) | 0.87 (0.29, 1.44) * | **0.004** | **0.039** | 1.11 (0.42, 1.79) | **0.002** | **0.035** |
| GV (m/s) | 1.11 (0.44, 1.78) | **0.002** | **0.030** | 1.31 (0.60, 2.01) | **0.001** | **0.014** |
| Stance phase | -13.54 (-22.73, -4.34) | **0.004** | **0.039** | -0.0002(-0.0001, -0.0006) * | **0.003** | **0.048** |
| Stride time * | -0.33 (-0.65, -0.01) | **0.043** | 0.164 | -0.41 (-0.89, -0.06) | 0.085 | 0.350 |
| Stance time * | -0.70 (-1.29, -0.10) | **0.023** | 0.142 | -0.94 (-1.85, -0.03) | **0.044** | 0.267 |
| Swing time | 0.01 (-0.09, 0.11) | 0.811 | 0.889 | -0.09 (-0.87, 0.69) | 0.820 | 0.930 |
| TO (°) * | 1382.88 (492.19, 2273.56) | **0.003** | **0.039** | 1691.66 (806.23, 2566.10) | **<0.001** | **0.014** |
| HS (°) | 30.65 (7.92, 53.37) | **0.009** | 0.076 | 30.27 (7.43, 53.11) | **0.010** | 0.136 |
| CV-GV (%) * | -0.04 (-0.15, 0.06) | 0.404 | 0.614 | -0.02 (-0.05, 0.01) | 0.125 | 0.418 |
| CV-SL (%) * | -0.04 (-0.08, 0.01) | 0.086 | 0.234 | -0.06 (-0.11, -0.002) | **0.043** | 0.267 |
| AI-SL (%) * | -1.34 (-2.86, 0.17) | 0.081 | 0.225 | -0.50 (-1.00, -0.01) | **0.048** | 0.267 |
| CV-stride time (%) * | 0.01 (-0.01, 0.02) | 0.373 | 0.604 | -0.02 (-0.11, 0.07) | 0.648 | 0.856 |
| AI-stride time (%) * | 0.12 (-0.06, 0.30) | 0.181 | 0.373 | -0.75 (-2.84, 1.33) | 0.474 | 0.806 |
| CV-swing time (%) * | -0.002 (-0.01, 0.01) | 0.724 | 0.847 | -0.001 (-0.01, 0.002) | 0.412 | 0.768 |
| AI-swing time (%) * | -0.04 (-0.58, 0.65) | 0.911 | 0.944 | -0.05 (-0.13, 0.04) | 0.268 | 0.661 |
| CV-stance time (%) * | 0.002 (-0.01, 0.01) | 0.729 | 0.847 | -0.0001 (-0.003, 0.003) | 0.934 | 0.957 |
| AI-stance time (%) * | 0.20 (-0.49, 0.90) | 0.079 | 0.224 | -0.07 (-0.21, 0.08) | 0.362 | 0.766 |

Multiple linear regression was used to examine the age- and sex-adjusted associations between gait parameters, and the values of GMD. *, indicated the residuals of multiple linear regression models were not normally distributed, and data transformation was performed. *p*’, *p* values corrected by Benjamini-Hochberg multiple testing correction for 136 comparisons. Bold values indicate statistical significance, *p, p’* ≤ 0.05.

Abbreviations: Ch123: cholinergic nuclei 123; PD: Parkinson’s disease; GMD: gray matter density; ST: single task; DT: dual task; SL: stride length; GV: gait velocity; TO: toe-off angle; HS: heel strike angle; CV: coefficient of variation; AI: asymmetry index.

|  | ST | | | DT | | |
| --- | --- | --- | --- | --- | --- | --- |
|  | B (95%CI) | *p* | *p*’ | B (95%CI) | *p* | *p*’ |
| **Left Ch4 GMV** |  |  |  |  |  |  |
| SL (m) | 0.01 (0.002, 0.03) * | **0.025** | 0.142 | 0.01 (-0.003, 0.03) | 0.053 | 0.267 |
| GV (m/s) | 0.02 (0.005, 0.03) | **0.011** | 0.079 | 0.02 (0.002, 0.03) | **0.028** | 0.248 |
| Stance phase | -0.21 (-0.41, -0.01) | **0.039** | 0.164 | -0.0002 (-0.0004, 0.0001) * | 0.072 | 0.316 |
| Stride time * | -0.01 (-0.013, 0.001) | 0.102 | 0.250 | -0.01 (-0.02, 0.004) | 0.281 | 0.661 |
| Stance time * | -0.01 (-0.03, 0.001) | 0.071 | 0.210 | -0.01 (-0.03, 0.01) | 0.226 | 0.591 |
| Swing time | -0.0002 (-0.002, 0.002) | 0.881 | 0.929 | -0.002 (-0.02, 0.01) | 0.833 | 0.930 |
| TO (°) * | 21.56 (2.06, 41.06) | **0.031** | 0.150 | 21.60 (1.73, 41.46) | **0.034** | 0.257 |
| HS (°) | 0.33 (-0.16, 0.82) | 0.187 | 0.374 | 0.22 (-0.28, 0.71) | 0.388 | 0.766 |
| CV-GV (%) * | -0.0002 (-0.002, 0.002) | 0.983 | 0.987 | 0.0004 (-0.001, 0.001) | 0.826 | 0.930 |
| CV-SL (%) * | -0.0001 (-0.001, 0.001) | 0.340 | 0.571 | -0.0001 (-0.001, 0.001) | 0.998 | 0.998 |
| AI-SL (%) * | -0.01 (-0.05, 0.02) | 0.411 | 0.614 | 0.003 (-0.01, 0.10) | 0.589 | 0.831 |
| CV-stride time (%) * | -0.0001 (-0.0001, 0.0001) | 0.810 | 0.889 | -0.0001 (-0.002, 0.002) | 0.889 | 0.930 |
| AI-stride time (%) * | -0.001 (-0.004, 0.003) | 0.761 | 0.865 | -0.01 (-0.06, 0.03) | 0.625 | 0.842 |
| CV-swing time (%) * | 0.0001 (-0.0001, 0.0001) | 0.761 | 0.865 | 0.0001 (-0.0001, 0.0005) | 0.876 | 0.930 |
| AI-swing time (%) * | 0.003(-0.01, 0.02) | 0.651 | 0.817 | -0.0003 (-0.002, 0.001) | 0.594 | 0.831 |
| CV-stance time (%) * | 0.0001 (-0.0001, 0.0001) | 0.506 | 0.708 | 0.0001 (-0.0002, 0.0004) | 0.527 | 0.831 |
| AI-stance time (%) * | 0.01 (-0.01 - 0.02) | 0.369 | 0.604 | -0.0004 (-0.003, 0.003) | 0.765 | 0.889 |
| **Right Ch4 GMV** |  |  |  |  |  |  |
| SL (m) | 0.01 (0.01, 0.02) * | **0.002** | **0.030** | 0.01 (0.003, 0.03) | **0.014** | 0.170 |
| GV (m/s) | 0.02 (0.01, 0.03) | **0.001** | **0.023** | 0.01 (0.001, 0.03) | **0.031** | 0.248 |
| Stance phase | -0.21 (-0.35, -0.07) | **0.004** | **0.039** | -0.0002 (-0.0004, 0.0001) * | 0.092 | 0.361 |
| Stride time * | -0.01 (-0.01, -0.0001) | 0.060 | 0.190 | -0.002 (-0.01, 0.01) | 0.516 | 0.826 |
| Stance time * | -0.01 (-0.02, -0.001) | **0.032** | 0.150 | -0.01 (-0.02, 0.01) | 0.410 | 0.768 |
| Swing time | 0.0004 (-0.001, 0.002) | 0.581 | 0.762 | 0.003 (-0.01, 0.02) | 0.620 | 0.842 |
| TO (°) * | 21.86 (8.21, 35.51) | **0.002** | **0.030** | 18.64 (3.77, 33.51) | **0.015** | 0.170 |
| HS (°) | 0.46 (0.11, 0.80) | **0.010** | 0.076 | 0.30 (-0.07, 0.67) | 0.111 | 0.401 |
| CV-GV (%) * | -0.001 (-0.003, 0.0001) | 0.123 | 0.284 | -0.0002 (-0.001, 0.0003) | 0.691 | 0.862 |
| CV-SL (%) * | -0.001 (-0.001, -0.0001) | **0.045** | 0.164 | -0.0002 (-0.001, 0.0001) | 0.280 | 0.661 |
| AI-SL (%) * | -0.02 (-0.05, -0.0001) | **0.045** | 0.164 | -0.006 (-0.01, 0.002) | 0.116 | 0.405 |
| CV-stride time (%) * | -0.0001 (-0.0001, 0.0001) | 0.296 | 0.518 | -0.0003 (-0.002, 0.001) | 0.682 | 0.862 |
| AI-stride time (%) * | -0.001 (-0.004, 0.002) | 0.402 | 0.614 | -0.01 (-0.04, 0.02) | 0.554 | 0.831 |
| CV-swing time (%) * | -0.0001 (-0.0001, 0.0001) | 0.176 | 0.368 | -0.0009 (-0.0005, 0.0003) | 0.729 | 0.877 |
| AI-swing time (%) * | -0.004 (-0.01, 0.01) | 0.441 | 0.645 | -0.001(-0.002, 0.001) | 0.375 | 0.766 |
| CV-stance time (%) * | -0.0001 (-0.0001, 0.0001) | 0.362 | 0.600 | 0.0001 (-0.0003, 0.0003) | 0.873 | 0.930 |
| AI-stance time (%) * | -0.002 (-0.01, 0.01) | 0.712 | 0.847 | -0.001 (-0.003, 0.002) | 0.537 | 0.831 |

**Supplemental Table 10.** Association between gait parameters and GMV of Ch4 in PD patient

Multiple linear regression was used to examine the age-, sex- and total intracranial volume adjusted associations between gait parameters and the values of GMV. *, indicated the residuals of multiple linear regression models were not normally distributed, and data transformation was performed. *p*’, *p* values corrected by Benjamini-Hochberg multiple testing correction for 136 comparisons. Bold values indicate statistical significance, *p, p’* ≤ 0.05.

Abbreviations: Ch4: cholinergic nuclei 4; PD: Parkinson’s disease; GMD: gray matter density; ST: single task; DT: dual task; SL: stride length; GV: gait velocity; TO: toe-off angle; HS: heel strike angle; CV: coefficient of variation; AI: asymmetry index.

**Supplemental Table 11**. Association between gait parameters and GMV of Ch123 in PD patients

|  | ST | | | DT | | |
| --- | --- | --- | --- | --- | --- | --- |
|  | B (95%CI) | *p* | *p*’ | B (95%CI) | *p* | *p*’ |
| **Left Ch123 GMV** |  |  |  |  |  |  |
| SL (m) | 0.01 (-0.004, 0.01) * | 0.240 | 0.453 | 0.002 (-0.01, 0.01) | 0.690 | 0.862 |
| GV (m/s) | 0.01 (-0.0002, 0.02) | 0.055 | 0.186 | 0.003 (-0.01, 0.02) | 0.580 | 0.831 |
| Stance phase | -0.14 (-0.29, -0.003) | **0.045** | 0.164 | 0.0002 (-0.0002, 0.0002) * | 0.877 | 0.930 |
| Stride time * | -0.01 (-0.01, -0.0001) | **0.046** | 0.164 | -0.0001 (-0.01, 0.01) | 0.936 | 0.957 |
| Stance time * | -0.01 (-0.02, -0.001) | **0.038** | 0.164 | 0.0002 (-0.01, 0.01) | 0.991 | 0.998 |
| Swing time | -0.001 (-0.002, 0.001) | 0.397 | 0.614 | -0.001 (-0.01, 0.01) | 0.874 | 0.930 |
| TO (°) * | 11.03 (-2.88, 24.93) | 0.118 | 0.279 | 6.28 (-8.52, 21.08) | 0.400 | 0.766 |
| HS (°) | 0.26 (-0.09, 0.60) | 0.142 | 0.317 | 0.06 (-0.30, 0.43) | 0.726 | 0.877 |
| CV-GV (%) * | -0.001 (-0.002, 0.001) | 0.510 | 0.708 | 0.0003 (-0.0002, 0.001) | 0.223 | 0.591 |
| CV-SL (%) * | -0.0001 (-0.001, 0.0001) | 0.499 | 0.707 | 0.0002 (-0.001, 0.001) | 0.393 | 0.766 |
| AI-SL (%) * | -0.006 (-0.03, 0.02) | 0.606 | 0.778 | 0.003 (-0.01, 0.01) | 0.501 | 0.826 |
| CV-stride time (%) * | -0.0001 (-0.0001, 0.0001) | 0.849 | 0.909 | 0.001 (-0.0004, 0.003) | 0.072 | 0.316 |
| AI-stride time (%) * | -0.001 (-0.002, 0.004) | 0.550 | 0.748 | 0.02 (-0.01, 0.05) | 0.187 | 0.541 |
| CV-swing time (%) * | -0.0001 (-0.0001, 0.0001) | 0.792 | 0.883 | 0.0004 (-0.0001, 0.0008) | 0.078 | 0.332 |
| AI-swing time (%) * | 0.01 (-0.004, 0.01) | 0.297 | 0.518 | 0.001 (0.0009, 0.002) | 0.133 | 0.421 |
| CV-stance time (%) * | 0.0001 (-0.0001, 0.0001) | 0.583 | 0.762 | 0.0002 (-0.0001, 0.0005) | 0.051 | 0.267 |
| AI-stance time (%) * | 0.01 (-0.003, 0.02) | 0.158 | 0.347 | 0.001 (-0.001, 0.004) | 0.171 | 0.506 |
| **Right Ch123 GMV** |  |  |  |  |  |  |
| SL (m) * | 0.01 (0.01, 0.02) | **<0.001** | **0.009** | 0.01 (0.01, 0.02) | **<0.001** | **0.014** |
| GV (m/s) | 0.01 (0.01, 0.02) | **<0.001** | **0.009** | 0.01 (0.01, 0.02) | **<0.001** | **0.014** |
| Stance phase | -0.18 (-0.28, -0.08) | **<0.001** | **0.016** | -0.0002 (-0.0003, -0.0007) | **0.004** | 0.060 |
| Stride time * | -0.003 (-0.01, -0.0003) | **0.060** | 0.190 | -0.003 (-0.01, 0.002) | 0.247 | 0.622 |
| Stance time * | -0.01 (-0.02, -0.001) | **0.021** | 0.136 | -0.01 (-0.02, 0.002) | 0.127 | 0.418 |
| Swing time | 0.001 (-0.0005, 0.002) | 0.277 | 0.513 | 0.002(-0.01, 0.01) | 0.562 | 0.831 |
| TO (°) * | 18.59 (9.01, 28.18) | **<0.001** | **0.009** | 20.72 (10.98, 30.46) | **<0.001** | **0.010** |
| HS (°) | 0.45 (0.21, 0.69) | **<0.001** | **0.010** | 0.42 (0.17, 0.66) | **0.001** | **0.023** |
| CV-GV (%) * | -0.001 (-0.002, 0.001) | 0.289 | 0.518 | -0.0003 (-0.001, 0.0004) | 0.157 | 0.485 |
| CV-SL (%) * | -0.001 (-0.001, -0.0001) | **0.028** | 0.150 | -0.001 (-0.001, -0.0001) | 0.051 | 0.267 |
| AI-SL (%) * | -0.02 (-0.04, -0.003) | **0.024** | 0.142 | -0.006 (-0.011, -0.0009) | **0.041** | 0.267 |
| CV-stride time (%) * | 0.0001 (-0.0001, 0.0001) | 0.391 | 0.614 | -0.0008 (-0.001, 0.001) | 0.889 | 0.930 |
| AI-stride time (%) * | 0.001 (-0.001, 0.003) | 0.279 | 0.513 | -0.01 (-0.03, 0.02) | 0.643 | 0.856 |
| CV-swing time (%) * | -0.0001 (-0.0001, 0.0001) | 0.612 | 0.778 | -0.0001 (-0.0004, 0.0002) | 0.748 | 0.877 |
| AI-swing time (%) * | -0.001 (-0.01, 0.01) | 0.843 | 0.909 | -0.0003 (-0.001, 0.001) | 0.456 | 0.806 |
| CV-stance time (%) * | 0.0001 (-0.0001, 0.0001) | 0.763 | 0.865 | 0.0001 (-0.0002, 0.0002) | 0.661 | 0.862 |
| AI-stance time (%) * | 0.002 (-0.01, 0.0001) | 0.119 | 0.279 | -0.0001 (-0.002, 0.001) | 0.590 | 0.831 |

Multiple linear regression was used to examine the age-, sex- and total intracranial volume adjusted associations between gait parameters and the values of GMV. *, indicated the residuals of multiple linear regression models were not normally distributed, and data transformation was performed. *p*’, *p* values corrected by Benjamini-Hochberg multiple testing correction for 136 comparisons. Bold values indicate statistical significance, *p, p’* ≤ 0.05.

Abbreviations: Ch4: cholinergic nuclei 4; PD: Parkinson’s disease; GMD: gray matter density; ST: single task; DT: dual task; SL: stride length; GV: gait velocity; TO: toe-off angle; HS: heel strike angle; CV: coefficient of variation; AI: asymmetry index.

**Supplemental Table 12**. Association between gait parameters and structural signatures of Ch123/Ch4 in PD patients adjusted for LEDD.

|  | B (95%CI) | *p* | *p’* |
| --- | --- | --- | --- |
| **Right Ch4 GMD** |  |  |  |
| ST-GV (m/s) | 1.49 (0.57, 2.42) | 0.002 | 0.003 |
| **Right Ch123 GMD** |  |  |  |
| ST-SL (m) * | 1.50 (0.40, 2.60) | 0.009 | 0.010 |
| ST-GV (m/s) | 1.18 (0.50, 1.85) | 0.001 | 0.002 |
| ST-Stance phase | -15.46 (-24.87, -6.05) | 0.002 | 0.003 |
| ST-TO (°) * | 1.24 (-0.02, 2.5) | 0.059 | 0.059 |
| DT-SL | 0.98 (0.31, 1.64) | 0.005 | 0.006 |
| DT-GV | 1.33 (0.62, 2.04) | <0.001 | 0.002 |
| DT- Stance phase* | 0.0002 (-0.0001, -0.0006) | 0.002 | 0.003 |
| DT-TO* | 1.41 (0.26, 2.56) | 0.020 | 0.021 |
| **Right Ch4 GMV** |  |  |  |
| ST-SL (m) * | 0.01 (0.005, 0.02) | 0.002 | 0.003 |
| ST-GV (m/s) | 0.02 (0.01, 0.03) | 0.001 | 0.002 |
| ST-Stance phase | -0.21 (-0.35, -0.06) | 0.007 | 0.008 |
| ST-TO (°) * | 0.03 (0.01, 0.05) | 0.004 | 0.005 |
| **Right Ch123 GMV** |  |  |  |
| ST-SL (m) * | 0.01 (0.005, 0.02) | 0.001 | 0.002 |
| ST-GV (m/s) | 0.01 (0.00, 0.02) | <0.001 | 0.002 |
| ST-Stance phase | -0.19 (-0.30, -0.09) | <0.001 | 0.002 |
| ST-TO (°) * | 16.42 (6.36, 26.47) | 0.002 | 0.003 |
| ST-HS (°) | 0.42 (0.19, 0.66) | 0.001 | 0.002 |
| DT-SL | 0.01 (0.006, 0.02) | 0.001 | 0.002 |
| DT-GV | 0.02(0.01, 0.023) | <0.001 | 0.002 |
| DT-TO* | 18.41 (8.11, 28.72) | 0.001 | 0.002 |
| DT-HS | 0.382 (0.14, 0.62) | 0.002 | 0.003 |

Multiple linear regression was used to examine the age-, sex-, and LEDD adjusted associations between gait parameters, and the values of GMD, age-, sex-, LEDD and total intracranial volume-adjusted associations between gait parameters and the values of GMV. *, indicated the residuals of multiple linear regression models were not normally distributed, and data transformations were performed. *p’, p* values corrected by Benjamini-Hochberg multiple testing correction.

LEDD: Levodopa equivalent daily dose; Ch123/Ch4: cholinergic nuclei 123/ cholinergic nuclei 4; PD: Parkinson’s disease; GMD: gray matter density; ST: single task; SL: stride length; GV: gait velocity; TO: toe-off angle; HS: heel strike angle; ROM: range of motion; DT: dual-task; GMV: gray matter volume; CV: coefficient of variation; AI: asymmetry index.

**Supplemental Table 13**. Association between gait parameters and structural signatures of Ch123/Ch4 in PD patients adjust for cofounders

|  | Gait domain | B | Lower 95%CI | Upper 95%CI | *p* | *p*’ |
| --- | --- | --- | --- | --- | --- | --- |
| ***Right Ch4 GMD*** |  |  |  |  |  |  |
| ST-GV (m/s) | Pace | 2.31 | 0.88 | 3.74 | 0.002 | 0.008 |
| ***Right Ch123 GMD*** |  |  |  |  |  |  |
| ST-SL (m)* | Pace | 1.24 | 0.10 | 2.36 | 0.037 | 0.037 |
| ST-GV (m/s) | Pace | 0.99 | 0.42 | 1.56 | 0.001 | 0.008 |
| ST-Stance phase | Rhythm | -9.47 | -17.76 | -1.18 | 0.029 | 0.030 |
| DT-SL | Pace | 0.87 | 0.18 | 1.56 | 0.016 | 0.020 |
| DT-GV | Pace | 0.80 | 0.15 | 1.44 | 0.019 | 0.022 |
| DT- Stance phase* | Rhythm | -0.26 | -0.46 | -0.06 | 0.013 | 0.018 |
| DT-TO* | ROM | 1.73 | 0.70 | 2.76 | 0.002 | 0.008 |
| ***Right Ch4 GMV*** |  |  |  |  |  |  |
| ST-SL (m)* | Pace | 0.03 | 0.01 | 0.04 | 0.002 | 0.008 |
| ST-GV (m/s) | Pace | 0.01 | 0.003 | 0.02 | 0.013 | 0.018 |
| ST-Stance phase | Rhythm | -0.19 | -0.33 | -0.04 | 0.013 | 0.018 |
| ST-TO (°)* | ROM | 0.03 | 0.01 | 0.04 | 0.006 | 0.013 |
| ***Right Ch123 GMV*** |  |  |  |  |  |  |
| ST-SL (m)* | Pace | 0.02 | 0.01 | 0.03 | 0.003 | 0.011 |
| ST-GV (m/s) | Pace | 0.01 | 0.006 | 0.02 | 0.001 | 0.008 |
| ST-Stance phase | Rhythm | -0.14 | -0.23 | -0.04 | 0.006 | 0.013 |
| ST-TO (°)* | ROM | 0.02 | 0.002 | 0.03 | 0.029 | 0.030 |
| ST-HS (°) | ROM | 0.31 | 0.09 | 0.53 | 0.009 | 0.016 |
| DT-SL | Pace | 0.01 | 0.004 | 0.02 | 0.004 | 0.012 |
| DT-GV | Pace | 0.01 | 0.003 | 0.02 | 0.008 | 0.015 |
| DT-TO* | ROM | 0.02 | 0.006 | 0.03 | 0.005 | 0.013 |
| DT-HS | ROM | 0.29 | 0.06 | 0.51 | 0.015 | 0.020 |

Multiple linear regression(stepwise) was used to examine the age-, sex-, MDS-UPDRS III, MMSE, HAMD-24 and HAMA-24 scores adjusted associations between gait parameters, and the values of GMD, age-, sex-, MDS-UPDRS III, MMSE, HAMD-24 and HAMA-24 scores, and total intracranial volume-adjusted associations between gait parameters and the values of GMV. *, indicated the residuals of multiple linear regression models were not normally distributed, and data transformation was performed. *p’, p* values corrected by Benjamini-Hochberg multiple testing correction.

MMSE: Mini-Mental State Examination; HAMD-24: Hamilton Rating Scale for Depression-24; HAMA-24: Hamilton Anxiety Rating Scale-24; MDS-UPDRS III: Movement Disorder Society Unified Parkinson’s Disease Rating Scale part III; Ch123/Ch4: cholinergic nuclei 123/ cholinergic nuclei 4; PD: Parkinson’s disease; GMD: gray matter density; ST: single task; SL: stride length; GV: gait velocity; TO: toe-off angle; HS: heel strike angle; ROM: range of motion; DT: dual-task; GMV: gray matter volume; CV: coefficient of variation; AI: asymmetry index.

**Supplemental Table 14**. Association between gait parameters and structural signatures of Ch123/Ch4 in PD patients’ subgroups

|  | B (95%CI) | *p* | *p*’ |
| --- | --- | --- | --- |
| ***PD patients with non-FOG*** |  |  |  |
| *Right Ch123 GMD* |  |  |  |
| ST-SL (m) * | 1.05 (0.41, 1.69) | 0.002 | 0.040 |
| ST-GV (m/s) | 1.27 (0.54, 2.01) | 0.001 | 0.001 |
| DT-SL | 1.58 (0.83, 2.33) | <0.001 | <0.001 |
| DT-GV | 1.77 (1.01, 2.53) | <0.001 | <0.001 |
| DT- Stance phase * | -0.0002 (-0.0003, -0.0001) | 0.001 | 0.001 |
| DT-TO* | 2165.49 (1166.10, 3164.89) | <0.001 | <0.001 |
| *Right Ch123 GMV* |  |  |  |
| ST-SL (m) * | 0.02 (0.01, 0.02) | <0.001 | <0.001 |
| ST-GV (m/s) | 0.02 (0.01, 0.03) | <0.001 | <0.001 |
| ST-TO (°) * | 22.84 (12.23, 33.46) | <0.001 | <0.001 |
| ST-HS (°) | 0.55 (0.29, 0.81) | <0.001 | <0.001 |
| DT-SL | 0.02 (0.01, 0.03) | <0.001 | <0.001 |
| DT-GV | 0.02 (0.01, 0.03) | <0.001 | <0.001 |
| DT-TO * | 29.57 (19.25, 39.89) | <0.001 | <0.001 |
| DT-HS | 0.66 (0.40, 0.91) | <0.001 | <0.001 |
| ***Left onset PD*** |  |  |  |
| *Right Ch123 GMV* |  |  |  |
| ST-Stance phase | -0.26 (-0.41, -0.11) | 0.002 | 0.042 |
| ST-TO (°) * | 24.55 (10.17, 38.94) | 0.002 | 0.037 |
| DT-TO * | 20.26 (9.17, 31.36) | 0.001 | 0.013 |

Multiple linear regression was used to examine the age- and sex- adjusted associations between gait parameters, and the values of GMD, age-, sex-, and total intracranial volume-adjusted associations between gait parameters and the values of GMV in different PD subgroups. *, indicated the residuals of multiple linear regression models were not normally distributed, and data transformations were performed. *p’, p* values corrected by Benjamini-Hochberg multiple testing correction.

FOG: freezing of gait; Early stage PD, PD patients at HY stage 1-2.5; Ch123/Ch4: cholinergic nuclei 123/ cholinergic nuclei 4; PD: Parkinson’s disease; GMD: gray matter density; ST: single task; SL: stride length; GV: gait velocity; TO: toe-off angle; HS: heel strike angle; ROM: range of motion; DT: dual-task; GMV: gray matter volume; CV: coefficient of variation; AI: asymmetry index.

**Supplemental Table 15**. Association between gait parameters and structural signatures of Ch123/Ch4 in PD patients with HY stages 1-2

|  | B (95%CI) | *p* | *p’* |
| --- | --- | --- | --- |
| **Right Ch123 GMD** |  |  |  |
| ST-GV (m/s) | 0.78 (0.07, 1.48) | 0.037 | 0.211 |
| DT- Stance phase* | -0.000001 (-0.0001, -0.000006) | 0.036 | 0.206 |
| DT-TO* | 1362.53 (287.36, 2437.70) | 0.017 | 0.113 |
| **Right Ch4 GMV** |  |  |  |
| ST-SL (m) * | 0.01 (0.002, 0.02) | 0.029 | 0.193 |
| ST-GV (m/s) | 0.02 (0.002, 0.03) | 0.024 | 0.192 |
| ST-TO (°) * | 18.89 (0.72, 37.06) | 0.048 | 0.240 |
| **Right Ch123 GMV** |  |  |  |
| ST-SL (m) * | 0.01 (0.003, 0.02) | 0.011 | 0.120 |
| ST-GV (m/s) | 0.01 (0.003, 0.02) | 0.009 | 0.120 |
| ST-TO (°) * | 15.95 (4.10, 27.80) | 0.012 | 0.120 |
| ST-HS (°) | 0.40 (0.12, 0.67) | 0.008 | 0.120 |
| DT-SL | 0.01 (0.00001, 0.02) | 0.005 | 0.050 |
| DT-GV | 0.014 (0.01, 0.02) | 0.002 | **0.040** |
| DT-TO* | 21.37 (9.44, 33.31) | 0.001 | **0.040** |
| DT-HS | 0.44 (0.14, 0.73) | 0.007 | 0.056 |

Multiple linear regression was used to examine the age-, sex-, and LEDD adjusted associations between gait parameters, and the values of GMD, age-, sex-, LEDD and total intracranial volume-adjusted associations between gait parameters and the values of GMV. *, indicated the residuals of multiple linear regression models were not normally distributed, and data transformations were performed. *p’, p* values corrected by Benjamini-Hochberg multiple testing correction. Bold values indicate statistical significance, p’ ≤ 0.05.

HY stage: Hoehn and Yahr stage; Ch123/Ch4: cholinergic nuclei 123/ cholinergic nuclei 4; PD: Parkinson’s disease; GMD: gray matter density; ST: single task; SL: stride length; GV: gait velocity; TO: toe-off angle; HS: heel strike angle; ROM: range of motion; DT: dual-task; GMV: gray matter volume; CV: coefficient of variation; AI: asymmetry index.

**Supplemental Table 16**. Association between gait parameters and structural signatures of Ch123/Ch4 in PD patients with HY stages >2

|  | B (95%CI) | *p* | *p’* |
| --- | --- | --- | --- |
| **Left Ch4 GMD** |  |  |  |
| DT-TO* | 2900.33 (318.83, 5481.83) | 0.037 | 0.154 |
| **Left Ch123 GMD** |  |  |  |
| DT-TO* | 34.65 (2.56, 66.74) | 0.044 | 0.146 |
| **Right Ch4 GMD** |  |  |  |
| ST-Stance phase | -0.22 (-0.43, -0.01) | 0.047 | 0.134 |
| ST-TO (°) * | 2397.3 (398.36, 4398.23) | 0.026 | 0.095 |
| DT-TO (°) * | 2590.46 (460.26, 4720.66) | 0.025 | 0.154 |
| **Right Ch123 GMD** |  |  |  |
| ST-SL (m) * | 1.19 (0.38, 2.00) | 0.007 | **0.035** |
| ST-GV (m/s) | 1.43 (0.45, 2.41) | 0.007 | **0.035** |
| ST-Stance phase | -19.43 (-32.01, -6.86) | 0.005 | **0.035** |
| ST-TO (°) * | 1998.51 (678.63, 3348.39) | 0.007 | **0.035** |
| ST-HS (°) | 46.59 (12.67, 80.50) | 0.011 | **0.044** |
| DT-SL | 1.39 (0.33, 2.45) | 0.016 | 0.154 |
| DT-GV | 1.40 (0.10, 2.69) | 0.044 | 0.154 |
| DT-TO* | 2063.85 (635.02, 3492.68) | 0.009 | 0.154 |
| DT-HS | 40.18 (4.56, 75.80) | 0.036 | 0.154 |
| **Right Ch4 GMV** |  |  |  |
| ST-Stance phase | -0.22 (-0.43, -0.01) | 0.047 | 0.134 |
| ST-TO (°) * | 26.13 (3.71, 48.54) | 0.030 | 0.095 |
| DT-TO (°) * | 28.41 (4.92, 51.90) | 0.026 | 0.154 |
| **Right Ch123 GMV** |  |  |  |
| ST-SL (m) * | 0.01 (0.004, 0.02) | 0.007 | **0.035** |
| ST-GV (m/s) | 0.02 (0.01, 0.03) | 0.006 | **0.035** |
| ST-Stance phase | -0.216 (-0.35, -0.09) | 0.003 | **0.035** |
| ST-TO (°) * | 21.59 (7.02, 36.15) | 0.007 | **0.035** |
| ST-HS (°) | 0.51 (0.15, 0.87) | 0.010 | **0.044** |
| DT-SL | 0.02 (0.003, 0.03) | 0.019 | 0.154 |
| DT-GV | 0.02 (0.001, 0.03) | 0.049 | 0.154 |
| DT-TO* | 21.96 (6.52, 37.39) | 0.010 | 0.154 |
| DT-HS | 0.43 (0.04, 0.81) | 0.040 | 0.145 |

Multiple linear regression was used to examine the age-, sex-, and LEDD adjusted associations between gait parameters, and the values of GMD, age-, sex-, LEDD and total intracranial volume-adjusted associations between gait parameters and the values of GMV. *, indicated the residuals of multiple linear regression models were not normally distributed, and data transformations were performed. *p’, p* values corrected by Benjamini-Hochberg multiple testing correction.

HY stage: Hoehn and Yahr stage; Ch123/Ch4: cholinergic nuclei 123/ cholinergic nuclei 4; PD: Parkinson’s disease; GMD: gray matter density; ST: single task; SL: stride length; GV: gait velocity; TO: toe-off angle; HS: heel strike angle; ROM: range of motion; DT: dual-task; GMV: gray matter volume; CV: coefficient of variation; AI: asymmetry index.
